# Proteomic analysis of effects by x-rays and heavy ion in HeLa cells

**DOI:** 10.2478/raon-2013-0087

**Published:** 2014-04-25

**Authors:** Zhitong Bing, Guanghui Yang, Yanan Zhang, Fengling Wang, Caiyong Ye, Jintu Sun, Guangming Zhou, Lei Yang

**Affiliations:** 1 Institute of Modern Physics, Chinese Academy of Sciences, Lanzhou, China; 2 Graduate School of Chinese Academy of Sciences, Beijing, China; 3 Biochemistry and Molecular Laboratory, Medical College of Henan University, Henan, China

**Keywords:** X-rays, Carbon ion, 2D-LC-MS/MS, SILAC, Gene Ontology enrichment

## Abstract

**Background:**

Carbon ion therapy may be better against cancer than the effects of a photon beam. To investigate a biological advantage of carbon ion beam over X-rays, the radioresistant cell line HeLa cells were used. Radiation-induced changes in the biological processes were investigated post-irradiation at 1 h by a clinically relevant radiation dose (2 Gy X-ray and 2 Gy carbon beam). The differential expression proteins were collected for analysing biological effects.

**Materials and methods.:**

The radioresistant cell line Hela cells were used. In our study, the stable isotope labelling with amino acids (SILAC) method coupled with 2D-LC-LTQ Orbitrap mass spectrometry was applied to identity and quantify the differentially expressed proteins after irradiation. The Western blotting experiment was used to validate the data.

**Results:**

A total of 123 and 155 significantly changed proteins were evaluated with treatment of 2 Gy carbon and X-rays after radiation 1 h, respectively. These deregulated proteins were found to be mainly involved in several kinds of metabolism processes through Gene Ontology (GO) enrichment analysis. The two groups perform different response to different types of irradiation.

**Conclusions:**

The radioresistance of the cancer cells treated with 2 Gy X-rays irradiation may be largely due to glycolysis enhancement, while the greater killing effect of 2 Gy carbon may be due to unchanged glycolysis and decreased amino acid metabolism.

## Introduction

Radiotherapy is one of the most important treatments for many human cancers. The statistics showed that at least 50 percent of patients who suffered from cancer received radiotherapy during the course of their therapy. X-rays and carbon beams have been widely applied in radiotherapy. Although X-rays treatment is an effective modality for variety of human cancers, in certain cases it can provide poor results.^1^ Some studies report that heavy ion beam have obvious advantages over other radiotherapy mainly due to the spread out Bragg’s peaks (SOBP), which can cover tumors with biological equivalent dose distribution.^2^,^3^ Besides, heavy ion can also reduce oxygen enhancement ratio, decrease cell-cycle-dependent radiosensitivity, and induce more DNA double strand breaks that are not easily repaired.^4^–^6^ Although many studies focused on the different biological effects between heavy ion and X-rays, few have revealed the mechanism of their difference on the systems level. Therefore, understanding the mechanisms of radiation effect on cancer cells will contribute to the development of powerful therapeutics for treatment of cancer. In recent years, the tendency of researches on exploring the difference between low-LET (X-ray and gamma ray) and carbon beam are increasing in clinical application.^7^,^8^

In fact, radiation biological effectiveness (RBE) means not only the response of different DNA damage to different types of radiation, but also the combined effects of protein interaction regulation and protein differential expression. So, proteomic analysis will provide more information as well as novel insight into understanding the difference of the two types of radiation therapies. In this study, our aim is to provide systems level insight into understanding molecular mechanism of the cancer cells exposure to different types of radiation. Therefore, a precise quantitative measurement, stable isotope labelling with amino acids in cell culture (SILAC) in combination with two-dimensional liquid chromatography-tandem mass spectrometry (2D-LC-MS/MS) shotgun proteomics^9^–^12^, was employed to investigate the response of HeLa cells exposed to different types of 2 Gy radiation. And it has been widely accepted that quantitative proteomic analysis was a powerful tool for investigating the radiation effect.^13^–^16^

By Gene Ontology (GO) term enrichment analysis and Ingenuity Pathway Analysis (IPA), we found that the therapeutic differences between X-ray and heavy ion beam on killing effects, DNA damage and survival fraction in cancer cells might be largely due to their different metabolism processes.

## Materials and methods

### Sample preparation

Human cervical carcinoma HeLa cells (ATCC, CCL-2) were maintained in DMEM (Gibco) at 37°C in the 5% CO_2_ air-humidified incubator. Cells in control group were prepared by supplementing the growth medium with light ^12^C_6_^14^N_4_ L-arginine and ^12^C_6_^14^N_2_ L-lysine. Cells irradiated were maintained in heavy ^13^C_6_^15^N_4_ L-arginine and ^13^C_6_^15^N_2_ L-lysine supplemented medium. At least 7 subcultures were performed to obtain efficiently labelled cell populations. We separated the heavy labelled cells into 2 groups to be exposed to carbon ion and X-rays irradiation.

### Irradiation

HeLa cells were trypsinized, counted and seeded in 25 cm^2^ flasks at a density of 5×10^5^ cells/flask. After 48 h of incubation, sample 1 was irradiated at room temperature with 2 Gy of high-LET carbon beam with original energy of 165 MeV/u generated by the Heavy Ion Research Facility at Lanzhou (HIRFL, Institute of Modern Physics, Chinese Academy of Science). Sample 2 was irradiated at room temperature using RX650 X-Ray irradiator (Faxitron, Lincolnshire, IL, USA) at a dose of 2 Gy. The X-Ray generator at 200 kVp and 20 mA with 0.5 mm AI and 0.5 Cu filters. The dose rate was 1 Gy/min. Cells were returned to the incubator for further incubation.

### Trypsin digestion

All protein samples were extracted for MS analysis. Cells were scraped into 6 M urea and sonicated for 10 min at 4°C. After centrifugation for 30 min at 20,000g, the supernatants were collected and stored at −80°C. Protein concentrations were measured using the Bradford method.

Extracted protein samples from irradiated cells and control cells were combined at a 1:1 ratio. In short, 100 μg of protein mixture was dissolved in 6 M urea and 25 mM NH_4_HCO_3_ and reduced with 10 mM DTT for 1 h room at temperature. Samples were alkylated by 40 mM iodacetamide in the dark for 1 h at room temperature, and then 40 mM DTT was added to quench the iodacetamide for 1 h at room temperature. After diluting 8 M urea with 25 mM NH_4_HCO_3_ to 0.6 M, subsequently trypsin was added at a ratio of 1:40 and digested at 37°C for overnight. In order to completely fragmentate proteins, trypsin was added to at a ratio of 1:40 again and digested at 37°C for 8 h. At last, trypsin digestion was stopped by adding 1% formic acid.^17^

### 2D-LC-MS/MS analysis

The tryptic peptide mixtures were analyzed by 2D-LC coupled to a linear ion trap mass spectrometer LTQ-Orbitrap (Thermo Electron, San Jose, CA, USA). For each experiment, the peptide mixtures (from about 100 μg proteins) were pressure-loaded onto a biphasic silica capillary column (250 um id) packed with 3 cm of reverse phase C18 resin (SP-120-3-ODS-A, 3 mm, the Great Eur-Asia Sci&Tech Devolopment, Beijing, China) and 3 cm of strong cation exchange resin (Luma 5 um SCX 100A, Phenomenex, Torrance, CA, USA). The buffers used were 0.1% FA (buffer A), 80% ACN/0.1% FA (buffer B), and 600 mM ammonium acetate/5% ACN/0.1% FA (buffer C). After sample loading, the biphasic column was first desalted with buffer A and then eluted using a 10-step salt gradient ranging from 0 to 600 mM ammonium acetate. After each salt gradient, a gradient of buffer B ranging from 0 to 100% was applied. Step 1 consisted of a 100-min gradient from 0 to 100% buffer B. For steps 2–9, after equilibrating with buffer A for the first 3 min, X% buffer C was applied for 5 min, and peptides were eluted using a linear gradient as follows: 0–10% buffer B in 5 min, 10–45% buffer B in 77 min, 45–100% buffer B in 10 min and 100% buffer B for 10 min, followed by re-equilibration with buffer A for 10 min. The 5-min buffer C percentages (X) were 5, 10, 15, 20, 25, 35, 50, 75%. The gradient used in the final step contained 3 min of 100% buffer A, 20 min of 100% buffer C, a 5-min gradient from 0 to 10% buffer B, a 72-min gradient from 10 to 55% buffer B and a 5-min gradient from 55 to100% buffer B. Then 100% buffer B was applied for 5 min, followed by a 5-min elution with buffer A and another 10-min elution with buffer B. The effluent of the biphasic column in each case was directed into an in-house-packed 10 cm C18 analytical column (100 um id, SP-120-3-ODS-A, 3 mm) with a 3- to 5-um spray tip. The flow rate at the tip was maintained at about 500 nL/min. Nano-electrospray ionization was performed at a spray voltage of 1.9 kV and a heated capillary temperature of 170°C. The MS instrument was set to the data-dependent acquisition mode with dynamic exclusion turned on, and maximum ion injection time was set to 100 ms. One MS survey scan, with mass range 400–2000 m/z, was followed by five MS/MS scans.^18^ All tandem mass spectra were collected using a normalized collision energy (a setting of 35%), an isolation window of 2 Da, and 1 micro-scan. The XCalibur data system (ThermoElectron, Waltham, MA, USA) was used to control the HPLC solvent gradients and the application of MS scanning functions.

### Data analysis and bioinformatics

Peptides were identified using the MaxQuant software package^19^, version 1.3.0.5. MS/MS spectra were searched against the human international Protein Index (IPI) database (version 3.87), which was released on Sep 27,2011, and contains 91,464 protein sequences. Precursor mass tolerance was set to 20 ppm for the first search. For the main search, a 6ppm precursor mass tolerance was used. The maximum precursor ion charge state used for searching was 7. D2-carbamidomethylation of cysteines (59.0340 delta mass) was searched as a fixed modification and oxidation of methionines (15.999 delta mass), heavy L-arginine (10.0083 delta mass) and heavy L-lysine (8.0142 delta mass) were search as variable modifications. Enzyme specificity was set to trypsin and a maximum of two missed cleavages was allowed for searching. The target-decoy- based false discovery rate (FDR) for peptide and protein identification was set to 1% for peptides and proteins.^20^ Unmodified, oxidized methionine, deamidated asparagines, and N-terminally acetylated peptides were utilized for protein quantification.

Protein level information was obtained from the MaxQuant Protein Groups table. The proteins that identified as reverse or contaminants were removed from the result. All reported proteins were identified by two or more unique peptides and quantified with two or more ratio counts. Then, the data analysis was using the MaxQuant software program to generate an average normalized heavy/light ratio over three biological replicates, and significance B values were calculated using Perseus software.^21^ To determine significance, we used the cutoff of a significant B score of less than 0.05.

In this study, Ingenuity Pathway Analysis (IPA) (Ingenuity^®^ Systems, www.ingenuity.com) was applied to obtain information of relationship, biological mechanism, functions, and pathways of differentially regulated proteins. The fold change with log_2_ ratio and IPI accession number of deregulated proteins were submitted to IPA.

The DAVID Bioinformatics resource and Protein Analysis Through Evolution Relationships (PANTHER)^22^ classification system were used to identify enriched gene ontology (GO) terms in our dataset.^23^,^24^ GO terms assigned a Benjamini-Hochberg adjusted p-value of less than 0.05 by DAVID were deemed to be enriched over the background gene set.

### Western Blot analysis

Antibodies to lactate dehydrogenase A (LDHA) (sc-27230) were purchased from Santa Cruz Biotechnology and Anti-SCO1 (54653) antibody was purchased from Anaspec. Antibodies to AKT (9272) antibodies were purchased from Cell Signaling Technology.

Cells were collected and lysed in appropriate amounts of lysis buffer (Biyuntian, Nanjing, China). Samples were centrifuged at 10,000 g, 4°C for 15 min and the concentration of total protein was determined from the supernatants using BCA protein assay kit (Pierce, Rockford, IL, USA). Thereafter, samples were mixed with sample buffer (250 mM Tris HCl, 5% β-mercaptoethanol, 50% glycerol, 10% SDS, 0.5% bromophenol blue), boiled for 5 min and equal amounts of protein (30 μg) were separated with 10% SDS-PAGE gels (Bio-Rad, Tokyo, Japan). PVDF membranes (GE healthcare, Beijing, China) were rinsed in 100% methanol for 10 s and subsequently placed in transfer buffer (48 mM Tris, 39 mM Glycine, 0.037% SDS, 20% methanol) for 5 min. Blotting was performed at 120V for 1.5 h in a wet transfer instrument (Bio-Rad, Hercules, CA). The membranes were blocked for 1 h in blocking buffer (5% skim milk) and incubated with primary antibodies for 2 h. The membranes were then washed three times with PBS containing 0.1% Tween20 and incubated with secondary antibody for 1 h. Finally, following washing the membranes, protein bands were visualized using the enhanced chemiluminescence system (Amersham-Buchler, Braunschweig, Germany) and exposed to X-ray medical film (Kodak, Tokyo, Japan). The image analysis of western blots were using Photoshop CS5 software (Adobe).

### Colony formation assay

Cell survival was determined by conventional colony-formation assay. The irradiated cells were collected by trypsinization and resuspended in RPMI-1640 medium complemented with 10% FBS. Cell concentration was determined with a cell counter (Coulter, model Z1 with a 100 μm aperture tube). Cells were diluted with medium and seeded in 60-mm Petri dishes (3002 Falcon) to provide 10–100 colonies per dish. Dishes were incubated for 2 Gy X-ray and 2 Gy carbon for HeLa cell line respectively, then fixed with 10% formalin and stained with 1% methylene blue.^25^

### ATP level measurements

Following irradiation and subsequent incubation for 1 h at 37°C, cells were washed thoroughly with 0.9% sodium chloride solution, harvested by centrifugation, resuspended in distilled water and then lysed in ice water using an ultrasonic cell disrupter (Sonics, Newtown, CT, USA). Sonication was performed 4 times for 10 s each time with a 30 s pause between sonication bursts. Then, the lysate was boiled for 10 min in a boiling water bath, cell debris was removed by centrifugation at 4000 rpm for 10 min, and the ATP levels in the supernatant were measured using an ATP determination kit (Nanjing Jiancheng, Nanjing, China). The total protein concentration in the cell lysates was assayed using a BCA protein assay kit (Pierce, Rockford, IL, USA).

### Lactic acid level measurements

Lactate production was measured using an enzymatic kit (Nanjing Jiancheng) by following the manufacturer’s instruction. These results were normalized by cell counts. Briefly, NAD^+^ was added to media and stoichiometrically converted to NADH by lactate in the media. The levels of NADH were then quantified colorimetrically, as described by the manufacturer.

## Results

### Protein identification and quantification

**FIGURE 1.**
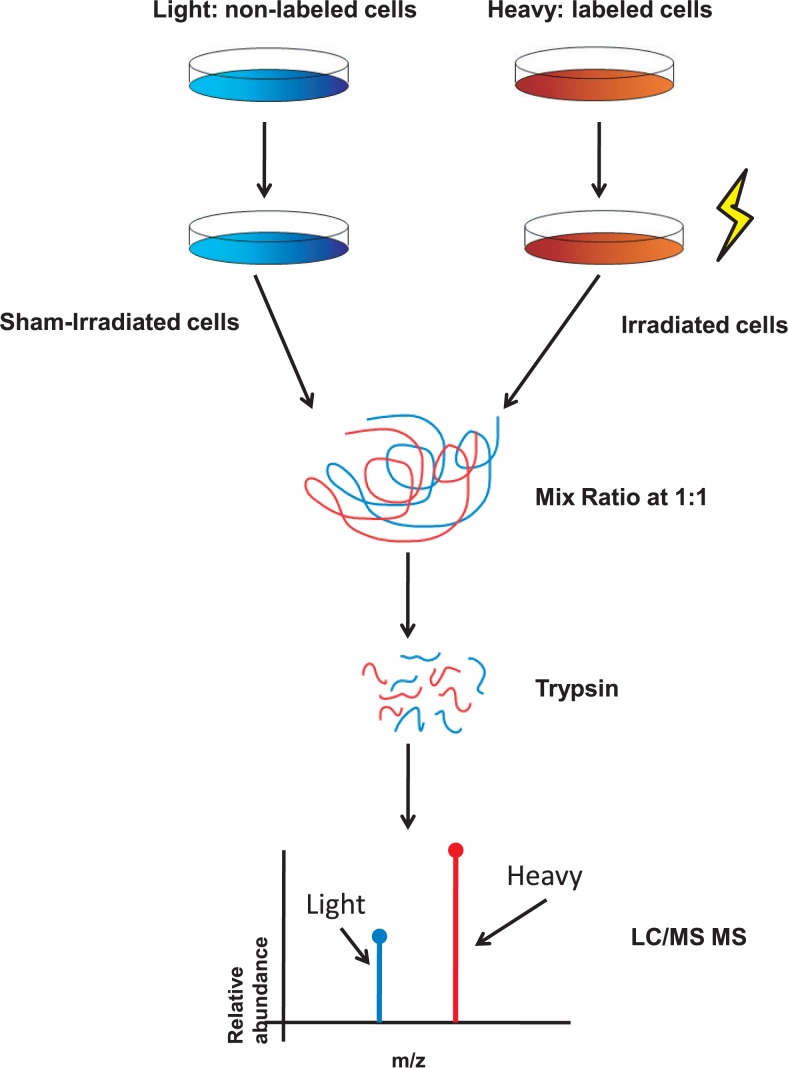
Brief SILAC experiment workflow. For the SILAC experiment, the HeLa cells were culture in DMEM containing “heavy” ^13^C_6_^15^N_4_ L-arginine, ^13^C_6_^15^N_2_ L-lysine and “light” ^12^C_6_^14^N_4_ L-arginine and ^12^C_6_^14^N_2_ L-lysine. After 100% label incorporation, heavy cells were irradiated and harvested after 1 h. Then, the cells were digested and mixed (treated *vs.* control) at 1:1 for mass spectrometric analysis.

HeLa cells were irradiated by 2 Gy carbon and X-rays respectively and then submitted to SILAC assay. The brief workflow was shown in Figure 1.

**FIGURE 2.**
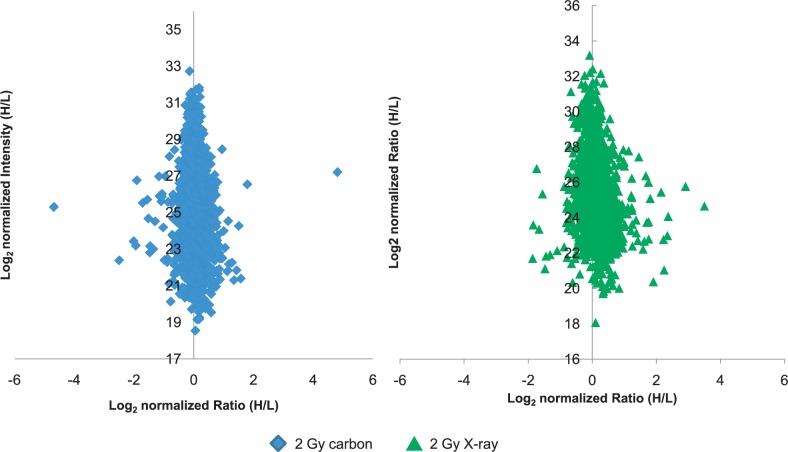
Distribution of log_2_ transformed protein expression ratios of two irradiation types. The proteins represented by data points lying close to the y-axis (y-axis=1) did not show any expression changes. Outliers were considered as proteins with significantly differential expression only if they had a p-value≤0.05 and were identified with a minimum of 2 unique peptides.

The analysis of three biological SILAC replicates was carried out upon two types of irradiation (2 Gy carbon and 2 Gy X-rays) at 1 h. In these samples, 1658 and 1627 proteins were quantified in 2 Gy carbon and 2 Gy X-ray, respectively. Of these, 123 and 155 proteins were significantly changed. In Figure 2, normalized protein ratios of all identified proteins by SILAC were plotted against summed peptide intensities. The data points lying close to the y-axis did not show any expression changes. Outliers were considered as proteins with differential expression only if they had significance B value ≤ 0.05 (the significance B score calculation see methods) and were identified with a minimum of 2 unique peptides. The fold changed threshold was set at ±1.3 and significance B value ≤ 0.05.

In this study, 123 and 155 deregulated proteins were quantified in 2 Gy carbon and 2 Gy X-rays, respectively (Supplementary File 1). A “Christmas tree” model representing normalised protein ratios of all identified proteins by SILAC plotted against summed peptide intensities is shown in Figure 2. In 2 Gy carbon group, 63 proteins were up-regulated and 60 were down-regulated. And in 2 Gy X-rays group, 76 proteins were up-regulated and 79 were down-regulated.

### The GO enrichment and Network analysis

**FIGURE 3.**
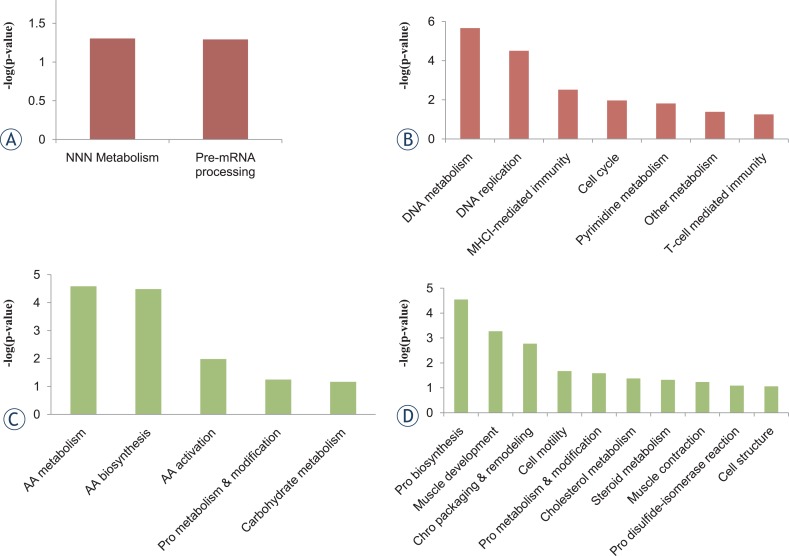
The biological process analysis for 2 Gy carbon and 2 Gy X-ray deregulated proteins. **(A)** the biological process distribution of up-regulated proteins in 2 Gy carbon irradiation(NNN, Nucleoside, nucleotide and nucleic acid). **(B)** The biological process distribution of up-regulated proteins in 2 Gy X-ray. C The biological process distribution of down-regulated proteins in 2 Gy carbon irradiation (AA, Amino acids; Pro, Protein;). **(D)** The biological process distribution of down-regulated proteins in 2 Gy X-ray (Chro, Chromatin; Pro, Protein).

The Gene Ontology (GO) enrichment of biological processes in two groups was done by searching database using PANTHER system. The analysis revealed some radiation-induced biological processes (see Supplementary File 2). The result of PANTHER system analysis indicated that the process catalogues of the deregulated proteins in two groups were very similar. Then, the up-regulated proteins and down-regulated proteins in two groups were respectively submitted to DAVID database for biological process detail analysis (Figure 3). Surprisingly, most of the proteins regulated biological processes from 2 Gy carbon group were quite different from 2 Gy X-rays.

In 2 Gy carbon, the up-regulated proteins were mainly involved in nucleotide metabolism and premRNA processing. While in 2 Gy X-ray, the up-regulated proteins were mainly involved in several kinds of metabolism, DNA repair and immunity. The distribution of up-regulated proteins in two groups indicated that HeLa cells might respond to irradiation through enhancing DNA metabolism. However, among the down-regulated proteins, it was found the distribution of down-regulated proteins in 2 Gy carbon was mainly involved in amino acid metabolism, which was a primary biological process with carbon beam irradiation. The down-regulated proteins in 2 Gy X-ray were involved in many biological processes.

To deeply understand the radiation response between the two groups, the protein interaction networks and pathway analysis were applied. The differentially expressed proteins of 2 Gy carbon and 2 Gy X-ray were submitted to IPA system respectively.

**FIGURE 4.**
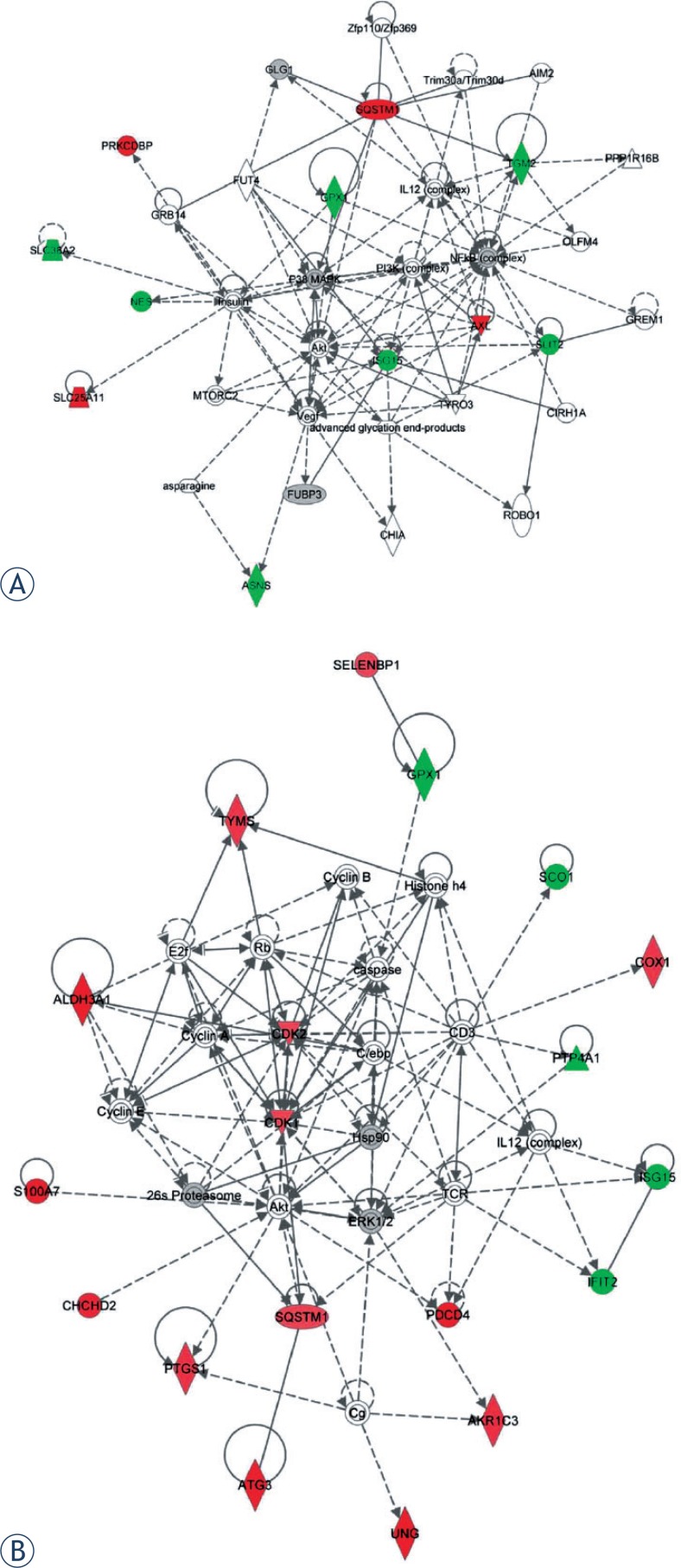
The highest score network post-irradiation by 2 Gy carbon and X-ray using IPA analysis. **(A)** The network “Cellular assembly and organization, cellular function and maintenance, post-translational modification, protein folding and cell death and survival” had a highest score of 29 post-irradiation by 2 Gy carbon. **(B)** The network “nucleic acid me tabolism, small molecule biochemistry, lipid metabolism, cellular assembly and organization, and DNA replication, recombination, and repair” had a highest score of 49 post-irradiation by 2 Gy X-ray. The shade of red represented significant up-regulated proteins and shade of green represented down-regulated proteins.

In 2 Gy carbon, the highest score network mainly involved in cellular functions of “Cellular Assembly and Organization, Cellular Function and Maintenance, Post-Translational Modification, Protein Folding and cell Death and Survival”. The network and proteins were showed in Table 1 and Figure 4A.

In 2 Gy X-ray, the deregulated proteins were involved in four protein networks. The most significant one with a score of 49 was involved in “nucleic acid metabolism, small molecule biochemistry, lipid metabolism, cellular assembly and organization, and DNA replication, recombination, and repair” (Figure 4B). These proteins involved in the network were indicated with their IPA names and log-ratio in Table 2.

**FIGURE 5.**
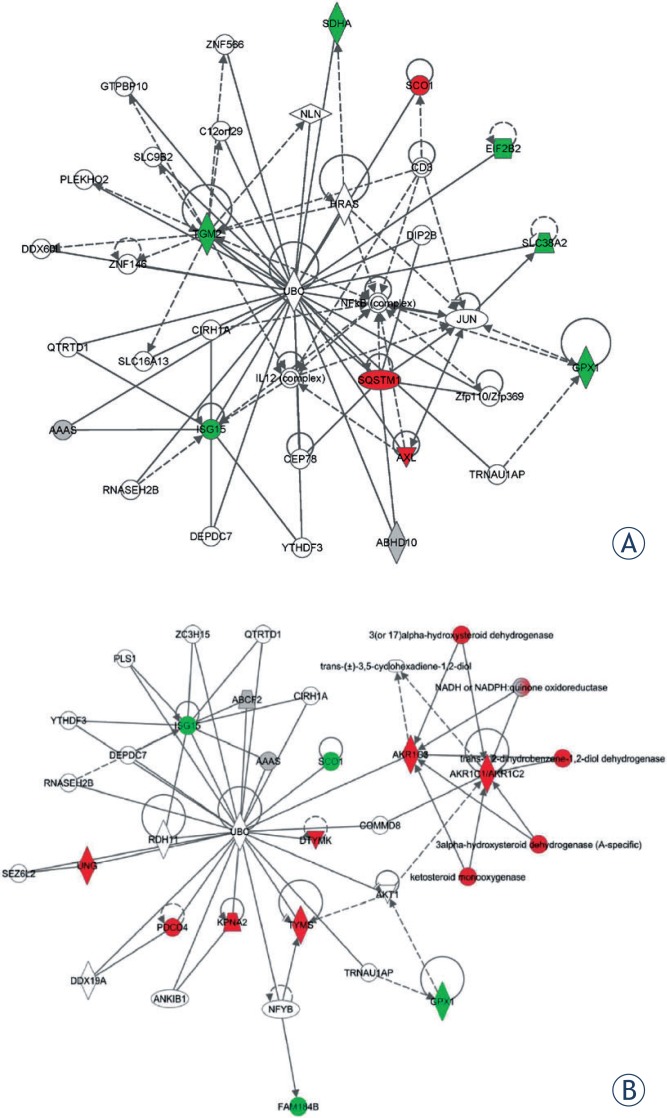
The highest score network of overlap deregulated proteins underlying 2 Gy carbon and X-ray using IPA analysis. **(A)** The overlap proteins with significant changes underlying 2 Gy carbon. The network “cellular assembly and organization, cellular function and maintenance, amino acid metabolism” had a highest score of 26. **(B)** The overlap proteins with significant changes underlying 2 Gy carbon. The network “lipid metabolism, small molecule biochemistry, nucleic acid metabolism” had a highest score of 32. (red: up-regulated; green: down-regulated).

To further understand the differential responses of two irradiation types, the overlapped proteins within two groups were separately submitted to IPA. Although the overlapped shared many common deregulated proteins, the distinction still existed between carbon beam and X-ray. In 2 Gy carbon, deregulated proteins network (Figure 5A) contained biological processes of “cellular assembly and organization, cellular function and maintenance, amino acid metabolism”, which might play a dominant role in response to carbon beam with a score of 26. As far as 2 Gy X-ray was concerned, the processes were mainly involved in “lipid metabolism, small molecule biochemistry, nucleic acid metabolism” with a score of 32 Figure 5B.

### Biological and function assay

**FIGURE 6.**
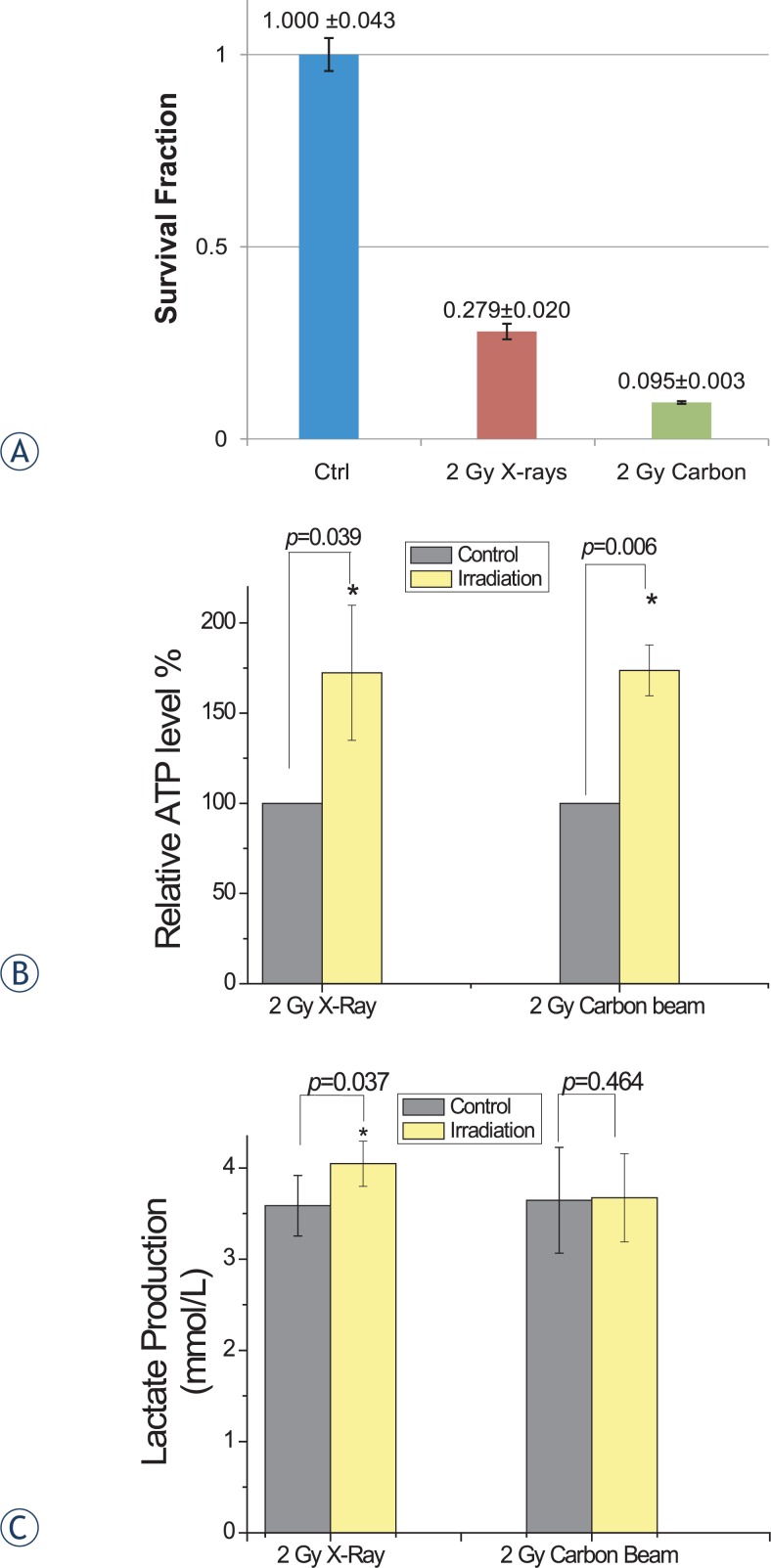
Biological and function assays for 2 Gy X-ray and carbon beam. **(A)** Survival fraction of HeLa cells after 2 Gy X-ray (red) or carbon beam (green) exposure. The plating efficiency of HeLa cells were 0.279±0.020 and 0.095±0.003 respectively in 2 Gy X-ray and 2 Gy carbon. **(B)** Changes in ATP levels between cells exposed to different irradiation and sham control (*, *p* < 0.05, t-test was one-tailed). (**C**) Differences in lactic acid levels between sham control and irradiated cells (*, *p* < 0.05, t-test was one-tailed).

To further validate the alternation of energy pathway by different types of irradiation, we assayed the survival fraction, ATP level and lactic acid level (Figure 6). As shown in Figure 6A, HeLa cells with 2 Gy X-ray had greater colony than the cells with 2 Gy carbon beam. The plating efficiency of HeLa cells were 0.279 ± 0.020 and 0.095 ± 0.003 respectively in 2 Gy X-ray and 2 Gy carbon. The Figure 6B showed that the ATP levels with two treatments were both up-regulated. Thus, the Figure 6C showed that the cells with 2 Gy X-ray treatment had higher lactic acid level than 2 Gy carbon beam.

### The MS data verification by Western Blotting

**FIGURE 7.**
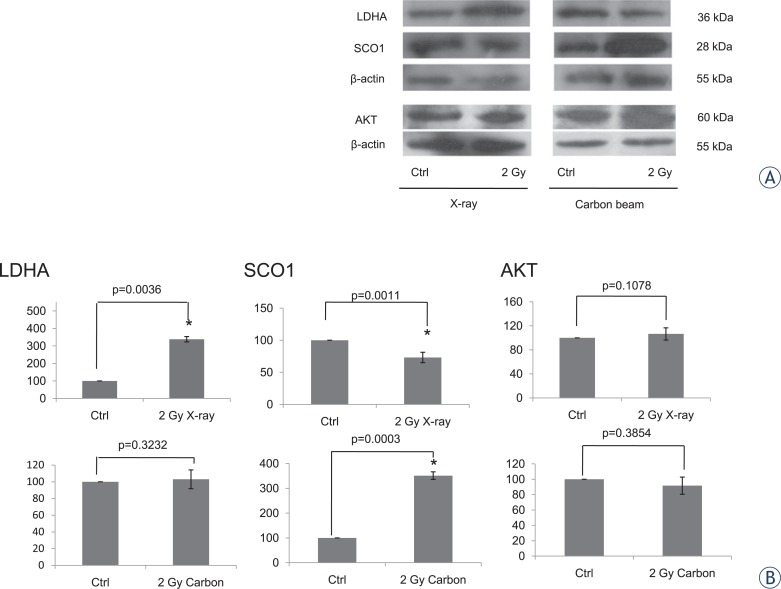
The western blotting for analysis of LDHA, SCO1 and Akt. **(A)** The images of western blot. **(B)** Fold changes between treated samples and control were analyzed by gray-values.

We found that the two types of radiation could activate different metabolism pathways. LDHA and SCO1 proteins represented glycolysis activation and oxidative phosphorylation activation respectively.^26^,^27^ So the two proteins were selected to verify MS data. The SILAC ratio of SCO1 was increased in 2 Gy carbon while decreased in 2 Gy X-rays. The SILAC ratio of LDHA was insignificantly changed in 2 Gy carbon but significantly elevated in 2 Gy X-rays. The expression changes of SCO1 and LDHA were confirmed by western blot analysis (Figure 7), which showed that the changes were basically identical with SILAC (Table 3).

## Discussion

In this study, we aimed to reveal the different cellular responses to the exposure of two irradiation types. In the previous study, it has been found that the survival fraction of cancer cells with carbon beam was lower than that of X-rays with same does, indicating that the carbon beam may have advantages over X-rays in radiotherapy.^2^,^3^,^28^ Although many studies have reported the changes of phenotype underlying different types of radiation, the molecular mechanism is still pending to be clarified to improve radiotherapy.

From deregulated proteins analysis, we found the two different irradiation types of 2 Gy carbon and 2 Gy X-ray demonstrated significant differences in cellular response (Figure 3). In 2 Gy carbon group, the result of IPA network analysis showed that the irradiation induced cell death and inhibiting cell growth. The up-regulated proteins Sequestosome1 (SQSTM1) and Protein kinase C delta-binding protein (PRKCDBP) were involved in promoting apoptosis.^29^,^30^ Although the up-regulated protein SCO1 was not directly involved in the regulated network (Figure 4A), the recent research has reported that SCO1 caused apoptosis by inducing reactive oxygen species in mitochondria.^31^ Furthermore, up-regulated mitochondrial proteins (SCO1, SLC25A11) and down-regulated GPX1 might indicate that the cancer cells were suffered from oxidation stress induced by 2 Gy carbon irradiation. Among down-regulated proteins, ASNS and SLC38A2 were closely associated with amino acid metabolism. ASNS was involved in asparagines synthesis and SLC38A2 was function as a sodium-dependent amino acid transporter.^32^,^33^ Other down-regulated proteins of IPA network such as GPX1, NES, TGM2 and SLIT2 were mainly involved in response to external stimulus and radiation by DAVID analysis (Supplementary File 3). The down-regulated protein ISG15 is an ubiquitin-like protein that involved in many biological processes. And we found ISG15 is regulated by many regulators from IPA analysis (Figure 4A). These down-regulated proteins indicated that the protein synthesis and response to radiation might be decreased. Additionally, some up-regulated proteins (SQSTM1, PRKCDBP and SCO1) were mainly involved in the apoptotic promotion. Many researchers reported the killing effect of heavy ion was stronger than X-ray due to DNA double strand breaks but hardly repair,^34^,^35^ which might be closely associated with many down-regulated proteins involved in amino acid metabolism process in 2 Gy carbon. Ra pid accumulation of biomass is necessary for cancer cell growth. When cancer cells are damaged by irradiation, the cells must generate enough energy and acquire or synthesize biomolecules at a sufficient rate to meet the demands of repair. Although we found ATP level increased (Figure 6B) in this study, the amino acid metabolism was not increased. As well known, amino acid metabolism plays an important role in biomass synthesis and most biomass are glycolytic intermediate production.^36^ Many enzymes down-regulated post-irradiation with 2 Gy carbon might lead to less biomass production and poor outcome of survival fraction. Thus, we inferred that the cancer cells might be more seriously damaged by 2 Gy carbon than 2 Gy X-ray.

In 2 Gy X-ray group, significant alternations were seen in the metabolic processes and DNA repair process (Figure 3 and Figure 6). Recent studies have reported that glycolysis and glucose utilisation were increased by radiation or oxidative stress.^37^–^39^ For example, glucose-6-phosphate dehydrogenase (G6PD), (pyruvate carboxylase) PC and (L-lactate dehydrogenase A) LDHA were up-regulated post-irradiation 1 h from mass spectrometry (see Supplement Table 1). In 2 Gy X-rays group, the up-regulated proteins in nucleic acid metabolism (Figure 3 and Figure 5B) were key proteins in the process of DNA damage repair. The results indicated that 2 Gy X-rays can enhance cell abilities of the DNA repair. Besides, in this study, we assayed the ATP level and lactic acid level increasing with 2 Gy X-ray. The increasing ATP level and lactic acid level indicated that the cells with 2 Gy X-ray generated energy mainly depending glycolysis. It has been reported that high level glycolysis would enhance capacity of radioresistance in cancer cells^40^,^41^, and the lactate from high level glycolysis might contribute to radioresistance. Because lactate production relies on reducing the pyruvate and this process recycles NADH back to NAD^+^, which would reduce the oxidative stress of irradiated cancer cells.^42^,^43^ That study also provide evidence that the poor outcome for patients with high lactate malignancies at least partially due to glycolysis-mediated resistance to radiotherapy.^42^ From IPA network results of 2 Gy X-ray, “nucleic acid metabolism, small molecule biochemistry, lipid metabolism, cellular assembly and organization, and DNA replication, recombination, and repair” were the most influenced biological pathway. From DAVID analysis of GO enrichment, among the up-regulated proteins, AKR1C3, COX1, PTGS1 and ALDH3A1were mainly involved in oxidation reduction. Besides, CDK1, CDK2, PDCD4, TYMS and UNG were mainly involved in cell cycle and response to DNA damage stimulus (Supplementary File 3). Other up-regulated proteins such as S100A7, CHCHD2, SELENBP1, ATG3 and SQSTM1 were not classified by DAVID. But we found S100A7, CHCHD2 and SQSTM1 directly interacted with Akt that plays a vital role in signalling pathway of cancer cells (Figure 4B). Previous study also found that ionizing radiation induced Akt activation in glioblastoma multiform, and the PI3KAkt signalling pathway has been correlated with radioresistance. Among the down-regulated proteins, the function of GPX1 and ISG15 were similar to 2 Gy carbon treatment. The rest down-regulated proteins SCO1, PTP4A1 and IFIT2 were associated with cell growth and apoptosis.^44^,^45^ In specific, down-regulation of SCO1 also indicated that oxidative phosphorylation process was decreased.

From the subsequent analysis of overlapped proteins network, we found metabolism process might play a vital role in irradiation treatment. Although the same proteins with differential expression were submitted to IPA, the network displayed distinct function modules. The Figure 5A showed that the regulation network of 2 Gy carbon treatment was mainly regulated by key nodal UBC, NF-κB complex, IL1 2 complex and JUN. Previous studies reported that NF-κB, IL12 and JUN were closely associated with ionizing radiation response.^46^–^48^ And Figure 5B showed that the regulation network of 2 Gy X-ray treatment was consisted of two modules that regulated by nodal UBC and AKR1 C1/AKR1C2. The two modules connected with nodal Akt, COMM D8 and AKR1C3. AKR1C1/AKR1C2 and AKR1C3 were involved in lipid metabolism. Akt is a key regulator for regulating many cell events and considered as a regulator of radioresistance.^49^

From the result of IPA network analysis (Figure 4 and Figure 5) and previous publication, increased energy metabolism might promote radioresistance and decreased amino acid might enhance radiosensitivity. We found that the different metabolism processes in response to irradiation might be associated with their survival fraction. Interestingly, we also found two networks with highest scores shared two regulators serine/threonine-specific protein kinase (Akt) and polyubiquitin-C (UBC). In this study, Akt and UBC proteins were not identified due to their very low abundance. Recent reports indicated that PTEN-PI3K/AKT pathway could regulate the cell death and cell cycle by ionizing radiation.^50^,^51^ Besides, Akt can also regulate energy metabolism.^52^,^53^ In this study, we assayed the Akt level with 2 Gy X-ray and 2 Gy carbon respectively. But we found that the Akt level was not significantly changed with two types of radiation treatment (Figure 7). In fact, different phosphorylation site of Akt would perform different function to regulate cell event. The regulation process of PI3KAkt and phosphorylation site need further study. To a great extent, the functions of UBC protein are decided by the different Lys residue modifications. In addition, the target proteins conjugating different sites of UBC might show distinct roles in biological processes. Therefore, the specific regulation mechanism of UBC protein was comparatively difficult to explore underling two irradiation types.

From above, we came to the conclusion that cancer cells in response to different types of radiation performed differently not only in DNA repair, but also in many other biological processes. Activation of the glycolysis, DNA metabolism and DNA repair process were seemed as key mechanisms for radioresistance of X-ray. Furthermore, previous study about metabolism of HeLa cells exposed to radiation reported some similar results with this study. Early in 1993, Karu *et al*. found ATP level of HeLa cells increased post-irradiation.^54^ And they provided four possibilities for explaining the reason of increasing ATP. But these possibilities have not been verified by experiment. In 2001, Grande *et al*. found that lactate of HeLa cells was increased 48 h after irradiation with high dose of gamma ray.^55^ Until recent years, with development of proteomic and genetic methods, the relationship between radiation and metabolism received more and more attention. Recent researches report that alternation of metabolism induced by radiation might be associated with cell damage and repair demand.^39^ And the result of distinct metabolism pathway induced by different types of radiation might provide some novel insight into improving clinical radiotherapy. At present, fluorine 18 fluorodeoxyglucose (FDG) positron emission tomography (PET) has been widely used for diagnosis, initial staging, and restaging of many kinds of cancer.^56^ The method could monitor glycolytic activity in malignant cells. And the distinct metabolism induced by radiation could be investigated by FDG-PET. We inferred that our finding integrating with FDG-PET could evaluate the effect of radiotherapy.

## Supplementary files

**Supplementary file 1.** List of all deregulated proteins underlying 2 Gy carbon and X-ray. The sheet 1 is table of 123 deregulated proteins underlying 2 Gy carbon beam. The sheet 2 is table of 155 deregulated proteins underlying 2 Gy X-ray. Available from: http://www.degruyter.com/view/j/raon.2014.48.issue-2/raon-2013-0087/suppl/raon-2013-0087_supp1.pdf

**Supplementary file 2.** Biological analysis of deregulated proteins by PANTHER system. Associated biological process of proteins found to be deregulated by 2 Gy carbon and 2 Gy X-ray irradiation. The differential expression proteins with significance were analyzed for biological processes using PANTHER classification system. Available from: http://www.degruyter.com/view/j/raon.2014.48.issue-2/raon-2013-0087/suppl/raon-2013-0087_supp2.pdf

**Supplementary file 3**. GO enrichment of deregulated proteins from IPA network. Many deregulated proteins that are not part of the results in 2 Gy carbon and 2 Gy X-ray are listed by GO enrichment from DAVID analysis. Available from: http://www.degruyter.com/view/j/raon.2014.48.issue-2/raon-2013-0087/suppl/raon-2013-0087_supp3.pdf

## Figures and Tables

**TABLE 1.** List of deregulated proteins in the highest score network after 2 Gy carbon irradiation

**Gene name**	**Protein name**	**IPI Acc**	**Log ratio**
SQSTM1	Sequestosome1	IPI00179473	1.799
PRKCDBP	Protein kinase C delta-binding protein	IPI00056334	1.582
SCO1	SCO cytochrome oxidase deficient homolog 1	IPI00027233	1.438
AXL	Tyrosine-protein kinase receptor UFO	IPI00296992	1.349
SLC25A11	**Mitochondrial 2-oxoglutarate/malate carrier protein**	IPI00219729	1.285
SLIT2	Slit homolog 2 protein	IPI00006288	−4.676
SDHA	Succinate dehydrogenase flavoprotein subunit, mitochondrial	IPI00305166	−2.494
SLC38A2	Sodium-coupled neutral amino acid transporter 2	IPI00410034	−2.012
EIF2B2	Translation initiation factor eIF-2B subunit beta	IPI00028083	−1.933
ISG15	Ubiquitin-like protein ISG15	IPI00375631	−1.903
NES	Nestin	IPI00010800	−1.700
FAM184B	Protein FAM184B	IPI00297208	−1.548
TGM2	Protein-glutamine gamma-glutamyltransferase2	IPI00218251	−1.513
PCNX	Pecanex-like protein 1	IPI00102678	−1.454
GPX1	Glutathione peroxidise 1	IPI00927606	−1.348

**TABLE 2.** List of deregulated proteins found in the highest score network after 2 Gy X-ray irradiation

**Gene name**	**Protein name**	**IPI Acc**	**Log ratio**
STK38l	Serine/threonine-protein kinase 38-like	IPI00237011	3.499
DTYMK	Thymidylate kinase	IPI00013862	2.371
SDHC	Succinate dehydrogenase cytochrome b560 subunit, mitochondrial	IPI00016968	2.346
ALDH3A1	Aldehyde dehydrogenase, dimeric NADP-preferring	IPI00296183	2.244
S100A7	Protein S100-7	IPI00219806	2.222
UNG	Uracil-DNA glycosylase	IPI00011069	1.907
PDCD4	Programmed cell death protein4	IPI00240675	1.807
ADI1	1,2-dihydroxy-3-keto-5-methylthiopentene dioxygenase	IPI00470791	1.762
KPNA2	Importin subunit alpha-2	IPI00002214	1.758
ATG3	Ubiquitin-like-conjugating enzyme ATG3	IPI00022254	1.756
DHX36	Probable ATP-dependent RNA helicase DHX36	IPI00027415	−1.863
NCSTN	Nicastrin	IPI00021983	−1.843
ISG15	**Ubiquitin-like protein ISG15**	IPI00375631	−1.734
IFIT2	Interferon-induce protein with tetratricopeptide repeats 2	IPI00018298	−1.664
FAM184B	Protein FAM184B	IPI00297208	−1.557
PTP4A1	Protein tyrosine phosphatise type IVA 1	IPI00020164	−1.479
SCO1	Protein SCO1 homolog, mitochondrial	IPI00027233	−1.441
GPX1	Glutathione peroxidise 1	IPI00927606	−1.318

**TABLE 3.** The Ratio comparison of SILAC and Western Blotting

**Gene name**	**SILAC Ratio (H/L)**		**Normalized Ratio (Western blot)**	
	**2 Gy Carbon**	**2 Gy X-ray**	**2 Gy Carbon**	**2 Gy X-ray**
LDHA	1.025 ± 0.13	1.829 ± 0.16^*^	1.030 ± 0.11	3.412 ± 0.82^*^
SCO1	1.438 ± 0.18^*^	0.368 ± 0.25^*^	3.513 ± 0.15^*^	0.760 ± 0.27^*^

* p-value≤0.05
