# Supplementary material for: Proteomic analysis of effects by x-rays and heavy ion in HeLa cells
Source: Radiol Oncol. 2014 Apr 25;48(2):142–54. doi: 10.2478/raon-2013-0087 (PMC4078033; doi:10.2478/raon-2013-0087)
Supplement: Supplementary file 3 [file raon-2014-issue2-0142_supp3.pdf]

2 Gy Carbon Beam

Category	Term		Count	%	PValue	Genes	List Total	Pop Hits	Pop Total	Fold Enrichment	Bonferroni	Benjamini	FDR
GOTERM_BP_FAT	GO:0032101	regulation of response to external stimulus	3	30	0,002773	GPX1, TGM2, SLIT2	8	159	13528	31,90566038	0,608858796	0,608858796	3,708087
GOTERM_BP_FAT	GO:0048514	blood vessel morphogenesis	3	30	0,004829	GPX1, TGM2, SLIT2	8	211	13528	24,04265403	0,805273198	0,558721401	6,373978
GOTERM_BP_FAT	GO:0043534	blood vessel endothelial cell migration	2	20	0,005679	GPX1, SLIT2	8	11	13528	307,4545455	0,85413465	0,473598183	7,456533
GOTERM_BP_FAT	GO:0001568	blood vessel development	3	30	0,00646	GPX1, TGM2, SLIT2	8	245	13528	20,70612245	0,888157638	0,421702144	8,440663
GOTERM_BP_FAT	GO:0001944	vasculature development	3	30	0,006771	GPX1, TGM2, SLIT2	8	251	13528	20,21115538	0,899383855	0,368267043	8,829691
GOTERM_BP_FAT	GO:0043542	endothelial cell migration	2	20	0,011331	GPX1, SLIT2	8	22	13528	153,7272727	0,978756627	0,473735695	14,36247
GOTERM_BP_FAT	GO:0009628	response to abiotic stimulus	3	30	0,014156	GPX1, NES, EIF2B2	8	368	13528	13,78532609	0,991925153	0,497635221	17,63288
GOTERM_BP_FAT	GO:0019725	cellular homeostasis	3	30	0,022166	GPX1, TGM2, EIF2B2	8	466	13528	10,88626609	0,999487518	0,612108878	26,28573
GOTERM_BP_FAT	GO:0010212	response to ionizing radiation	2	20	0,030643	GPX1, NES	8	60	13528	56,36666667	0,999972994	0,68926969	34,52163
GOTERM_BP_FAT	GO:0032103	positive regulation of response to external stimulus	2	20	0,032657	TGM2, SLIT2	8	64	13528	52,84375	0,999986629	0,674451325	36,3485
GOTERM_BP_FAT	GO:0042063	gliogenesis	2	20	0,03316	EIF2B2, SLIT2	8	65	13528	52,03076923	0,999988784	0,645199844	36,79728
GOTERM_BP_FAT	GO:0050727	regulation of inflammatory response	2	20	0,038678	GPX1, TGM2	8	76	13528	44,5	0,999998379	0,670786291	41,53191
GOTERM_BP_FAT	GO:0042592	homeostatic process	3	30	0,053654	GPX1, TGM2, EIF2B2	8	751	13528	6,754993342	0,999999992	0,761601907	52,7783
GOTERM_BP_FAT	GO:0001525	angiogenesis	2	20	0,07413	GPX1, SLIT2	8	148	13528	22,85135135	1	0,844250885	64,93393
GOTERM_BP_FAT	GO:0006732	coenzyme metabolic process	2	20	0,076549	SDHA, GPX1	8	153	13528	22,10457516	1	0,83379104	66,16051
GOTERM_BP_FAT	GO:0010740	positive regulation of protein kinase cascade	2	20	0,083296	GPX1, TGM2	8	167	13528	20,25149701	1	0,840745786	69,37359
GOTERM_BP_FAT	GO:0051186	cofactor metabolic process	2	20	0,096662	SDHA, GPX1	8	195	13528	17,34358974	1	0,86750586	74,92136
GOTERM_BP_FAT	GO:0009314	response to radiation	2	20	0,099032	GPX1, NES	8	200	13528	16,91	1	0,858893489	75,80162

2 Gy X-ray

Category	Term		Count	%	PValue	Genes	List Total	Pop Hits	Pop Total	Fold Enrichment	Bonferroni	Benjamini	FDR
GOTERM_BP_FAT	GO:0006693	prostaglandin metabolic process	2	14,28571	0,016731	AKR1C3, PTGS1	13	19	13528	109,5384615	0,988758282	0,988758282	19,85478
GOTERM_BP_FAT	GO:0006692	prostanoid metabolic process	2	14,28571	0,016731	AKR1C3, PTGS1	13	19	13528	109,5384615	0,988758282	0,988758282	19,85478
GOTERM_BP_FAT	GO:0055114	oxidation reduction	4	28,57143	0,016762	AKR1C3, COX1, PTGS1, ALDH3A1	13	639	13528	6,514024317	0,988851803	0,894414977	19,88779
GOTERM_BP_FAT	GO:0051726	regulation of cell cycle	3	21,42857	0,033504	CDK1, PDCD4, CDK2	13	331	13528	9,431559377	0,999884344	0,951278234	36,0476
GOTERM_BP_FAT	GO:0006690	icosanoid metabolic process	2	14,28571	0,04092	AKR1C3, PTGS1	13	47	13528	44,28150573	0,999985094	0,937864079	42,19348
GOTERM_BP_FAT	GO:0006974	response to DNA damage stimulus	3	21,42857	0,041692	CDK1, TYMS, UNG	13	373	13528	8,369560734	0,999987966	0,896227125	42,80041
GOTERM_BP_FAT	GO:0033559	unsaturated fatty acid metabolic process	2	14,28571	0,044331	AKR1C3, PTGS1	13	51	13528	40,80844646	0,999994221	0,86604174	44,83277
GOTERM_BP_FAT	GO:0006259	DNA metabolic process	3	21,42857	0,071926	TYMS, UNG, CDK2	13	506	13528	6,169656431	0,999999998	0,941366672	62,43694
GOTERM_BP_FAT	GO:0033554	cellular response to stress	3	21,42857	0,087404	CDK1, TYMS, UNG	13	566	13528	5,515629247	1	0,952219054	69,87382
GOTERM_BP_FAT	GO:0019941	modification-dependent protein catabolic process	3	21,42857	0,089543	CDK1, SQSTM1, ATG3	13	574	13528	5,438756366	1	0,937499142	70,78697
GOTERM_BP_FAT	GO:0043632	modification-dependent macromolecule catabolic process	3	21,42857	0,089543	CDK1, SQSTM1, ATG3	13	574	13528	5,438756366	1	0,937499142	70,78697
GOTERM_BP_FAT	GO:0007568	aging	2	14,28571	0,093364	PTGS1, PDCD4	13	110	13528	18,92027972	1	0,926257801	72,35485
GOTERM_BP_FAT	GO:0051603	proteolysis involved in cellular protein catabolic process	3	21,42857	0,096608	CDK1, SQSTM1, ATG3	13	600	13528	5,203076923	1	0,914295218	73,62469
GOTERM_BP_FAT	GO:0044257	cellular protein catabolic process	3	21,42857	0,097434	CDK1, SQSTM1, ATG3	13	603	13528	5,177190968	1	0,896934237	73,93931
