# Supplementary material for: Proteomic analysis of effects by x-rays and heavy ion in HeLa cells
Source: Radiol Oncol. 2014 Apr 25;48(2):142–54. doi: 10.2478/raon-2013-0087 (PMC4078033; doi:10.2478/raon-2013-0087)
Supplement: Supplementary file 1 [file raon-2014-issue2-0142_supp1.pdf]

2 Gy carbon beam 1 h SILAC LC-MS/MS

Uniprot Acc	Gene Name	Mod. peptide IDs	Peptide IDs	Peptide counts (all)	Peptide counts (razor+unique)	Peptide counts (unique)	Protein IDs
O94813-1	SLIT2	3437;6535	3319;6267	2;2;2;2	2;2;2;2	2;2;2;2	IPI00963814;IPI00006288;IPI00335003;IPI00220114
E9PBJ5	SDHA	34;111;254;1072;1864;2659;3149;3596;4530;5125;7813;7819;8711;9326;9372;9451	34;111;248;1032;1803;2574;3048;3466;4361;4936;7507;7511;8362;8952;8998;9075	1;1;1;0;0;0	1;1;1;0;0;0	0;0;0;0;0;0	IPI00964764;IPI00217143;IPI00902754;IPI00964725;IPI00956532;IPI00909676
Q96QD8-1	slc38a2	7614;8141;9763	7315;7821;9374	3;2;1	3;2;1	3;2;1	IPI00410034;IPI00789960;IPI01022723
P49770	EIF2B2	3757	3622	1;1;1	1;1;1	1;1;1	IPI00028083;IPI01025566;IPI01025442
P05161	ISG15	3852;4917;5820;6780;9321;9322	3712;4733;5608;6505;8947;8948	6	6	6	IPI00375631
P48681	NES	7728	7423	1;1	1;1	1;1	IPI00010800;IPI00844287
Q9ULE4	Fam184b	4140	3984	1	1	1	IPI00297208
P21980-1	tgm2	466;6013;9534;9597;9895	452;5792;9154;9216;9502	5;5;5;2;1	5;5;5;2;1	5;5;5;2;1	IPI00294578;IPI01013011;IPI01013460;IPI00218251;IPI00218252
O15143	ARPC1B	808;8700	777;8351	2;2;2;2;2;2;2;1;1;1	2;2;2;2;2;2;2;1;1;1	2;2;2;2;2;2;2;1;1;1	IPI00005160;IPI00927418;IPI00925033;IPI00924615;IPI00927612;IPI00927861;IPI00927206;IPI00925248;IPI00925800;IPI00924861
Q96RV3-1	pcnx	9354	8980	1;1;1;1;1	1;1;1;1;1	1;1;1;1;1	IPI00102678;IPI00375256;IPI01013985;IPI01025081;IPI01025421
P07203	GPX1	2931;10044	2838;9644	2	2	2	IPI00927606
P08243	asnS	155;2154;3810;4025;8905	153;2084;3673;3875;8550	5;5;4;3;2;1;1;1	5;5;4;3;2;1;1;1	5;5;4;3;2;1;1;1	IPI00554777;IPI00925572;IPI00924906;IPI00926480;IPI00925298;IPI00926959;IPI00926760;IPI00925986
P00966	ASS1	564;1047;1568;2072;2152;4778;6594	543;1007;1514;2006;2082;4601;6322	7;4;3;2;1	7;4;3;2;1	7;4;3;2;1	IPI00020632;IPI00909521;IPI00640430;IPI00642128;IPI00514341
O43175	PHGDH	270;503;3105;4023;6284;6929;8551	264;485;3005;3873;6027;6651;8211	7;6;5	7;6;5	7;6;5	IPI00011200;IPI00642548;IPI01010638
P25815	S100P	6220;8202;9997	5971;7879;9597	3	3	3	IPI00017526
P23381-1	wars	120;447;706;2805;2806;3601;3856;4196;4412;6197;6742;6802;7193;8165;9643	120;434;682;2714;2715;3471;3715;4039;4248;5953;6468;6527;6909;7845;9259	15;14;7;6;6;6;5;5;5;4;4;4;2;2;2;2;2;1;1;1;1;1;1;1;1;1;1;1	15;14;7;6;6;6;5;5;5;4;4;4;2;2;2;2;2;1;1;1;1;1;1;1;1;1;1;1	15;14;7;6;6;6;5;5;5;4;4;4;2;2;2;2;2;1;1;1;1;1;1;1;1;1;1;1	IPI00295400;IPI00412737;IPI01026281;IPI01025933;IPI01026173;IPI01024903;IPI01026146;IPI01024784;IPI01025786;IPI01025297;IPI01025791;IPI01025659;IPI01025287;IPI01026031;IPI01025395;IPI01026179;IPI01025496;IPI01025033;IPI01025032;IPI01026523;IPI01026267;IPI01026350;IPI01025155;IPI01025153;IPI01026283;IPI01024905;IPI01025693;IPI01026528
P09923	ALPI	532;2740;2958;6498;6532;8205;9347;9760	511;2650;2863;6231;6264;7881;8973;9371	5	5	5	IPI00298622
P32929-1	CTH	5106;5768;9103	4917;5558;8739	3;3;3	3;3;3	3;3;3	IPI00031557;IPI00908512;IPI00221301
Q5H9R7-5	SAPS3	3336;6880	3225;6603	2;2;2;2;2;2;2;2;2;1;1;1	2;2;2;2;2;2;2;2;2;1;1;1	2;2;2;2;2;2;2;2;2;1;1;1	IPI00719725;IPI01015850;IPI00719821;IPI00719553;IPI00030624;IPI00983215;IPI00985128;IPI00019540;IPI00719345;IPI00984704;IPI00975695;IPI00983893
Q9Y617-1	C8orf62	423;664;969;2210;2215;2594;3146;4455;5575;6694;7915	410;642;931;2140;2144;2513;3045;4288;5371;6421;7605	11;9;6	11;9;6	11;9;6	IPI00001734;IPI00219478;IPI01014011
P07951-2	tpm2	1527;4156;4642;5873;7220;7766;8336	1473;4000;4471;5659;6935;7461;8006	3;3;2;1;0;0;0	3;3;2;1;0;0;0	3;3;2;1;0;0;0	IPI00220709;IPI00218820;IPI00646748;CON__Q3SX28;IPI00513698;IPI00013991;IPI00910712
Q969X5-1	ERGIC1	2146;3270;5826;10015	2076;3163;5613;9615	4;4;2;2;2;2;2	4;4;2;2;2;2;2	4;4;2;2;2;2;2	IPI00022887;IPI00003635;IPI00916582;IPI00983506;IPI00021763;IPI00916999;IPI00916451
Q96C24-1	SYTL4	6811	6536	1;1;1	1;1;1	1;1;1	IPI00060201;IPI01015206;IPI00639966
Q14651	PLS1	799;5747;6330	771;5538;6070	1;1	1;1	1;1	IPI00032304;IPI00945930
P41250	Gars	177;1566;2414;4077;4078;5132;7494;7870;8230;8237;8480;9325;9964	174;1512;2337;3923;4943;7199;7560;7904;7911;8141;8951;9564	12	12	12	IPI00783097
G5E9P9	Rap1gds1	4106;6124	3951;5892	2;1;1;1;1;1;1;1;1	2;1;1;1;1;1;1;1;1	2;1;1;1;1;1;1;1;1	IPI00855727;IPI00855767;IPI00607591;IPI00619924;IPI00856100;IPI00424869;IPI00967154;IPI00967796;IPI00968047
Q01650	SLC7A5	403;7384;9636	391;7093;9253	3	3	3	IPI00008986
P14324	FDPS	903;5314;6163;6862;8125;9236	869;5119;5926;6585;7806;8867	6;6;4;2;1	6;6;4;2;1	6;6;4;2;1	IPI00914566;IPI00914971;IPI00902799;IPI00382869;IPI00455004
P07602-2	PSAP	1709;7462	1652;7168	2;2;2;2;2	2;2;2;2;2	2;2;2;2;2	IPI01219825;IPI00744835;IPI00873020;IPI00012503;IPI01013537
Q9GZM8-2	NDEL1	1751;4185	1694;4029	2;2;2;2;1;1	2;2;2;2;1;1	2;2;2;2;1;1	IPI00551002;IPI00384038;IPI00940808;IPI00006602;IPI00759601;IPI00014893
P11766	ADH5	97;8827	97;8474	2;1;1;1;1	2;1;1;1;1	2;1;1;1;1	IPI00746777;IPI00966735;IPI00964823;IPI00964589;IPI00967275
P06737	PYGL	1009;2579;5243;9634	970;2498;5048;9251	4;4;4	4;4;4	4;4;4	IPI00783313;IPI00977162;IPI00943894
P30740	SERPINB1	3482;8233;8713;8721	3358;7907;8364;8372	4;4;1	4;4;1	4;4;1	IPI00027444;IPI01014238;IPI01013899
Q96EE3-1	seh1l	1168;9353	1127;8979	2;2	2;2	2;2	IPI00220609;IPI00185533
Q16539-1	Mapk14	6162;9853	5925;9460	2;2;2;1;1;1	2;2;2;1;1;1	2;2;2;1;1;1	IPI00002857;IPI00221141;IPI00945296;IPI00221143;IPI00221142;IPI00984307
P08195-4	SLC3A2	146;938;974;975;1176;1992;2671;2928;3035;3812;5034;5325;5476;6607;7039;7756;7894;8745;9083;9691	144;901;936;937;1135;1928;2584;2835;2939;3675;4848;5128;5276;6335;6759;7451;7584;8395;8720;9305	20;20;20;20;20;20;20;7;5;4;2;2;1	20;20;20;20;20;20;20;7;5;4;2;1	20;20;20;20;20;20;20;7;5;4;2;1	IPI00554481;IPI01010400;IPI00604710;IPI00554611;IPI00554722;IPI00027493;IPI01013280;IPI01012329;IPI01010919;IPI00908885;IPI01014635;IPI01011854
P05413	fabp3	5273;7734	5078;7429	2	2	2	IPI00219684
Q13907-2	IDI1	608;9690	587;9304	2;2	2;2	2;2	IPI00220014;IPI00645307
Q9UH65	SWAP70	382;5675	371;5467	2;2;1	2;2;1	2;2;1	IPI00307200;IPI00903155;IPI00984765
Q7L1Q6	BZW1	2125;4466	2056;4299	2;2;2;2;1;1;1;1;1	2;2;2;2;1;1;1;1;1	2;2;2;2;1;1;1;1;1	IPI00916503;IPI00916112;IPI00785096;IPI00180128;IPI00917597;IPI00916463;IPI00915826;IPI00916338;IPI00916588
Q16719	Kynu	2477;2478;3731;4142;4698;5647;6743;7325;8042;8718;8719;8773;8785;9900	2399;2400;3596;3986;4524;5440;6469;7035;7729;8369;8370;8421;8432;9506	14;7;5;1	14;7;5;1	14;7;5;1	IPI00003818;IPI00651624;IPI00916732;IPI00915775
Q14139-2	ube4a	8248	7921	1;1;1	1;1;1	1;1;1	IPI00028957;IPI00556075;IPI00807340
P42785	prcp	3422;7572	3304;7274	2;2;2;2;2;2;2;1;1;1	2;2;2;2;2;2;2;1;1;1	2;2;2;2;2;2;2;1;1;1	IPI00399307;IPI00001593;IPI00980998;IPI00982987;IPI00983229;IPI00982909;IPI00981533;IPI00983889;IPI00976115;IPI00985278
Q14116	IL18	2251;6507	2180;6239	2;1	2;1	2;1	IPI00290198;IPI00980067
O60568	Plod3	4503;5806;8730;8956;9744	4334;5594;8381;8597;9355	5;3	5;3	5;3	IPI00030255;IPI00926874
Q8NBJ5	GLT25D1	1538;2662;6277;7803	1484;2577;6020;7497	4;1	4;1	4;1	IPI00168262;IPI00450971
P30533	Lrpap1	9030;9399	8671;9024	2	2	2	IPI00026848
O00592-1	PODXL	967;5305;9526	929;5110;9146	3;3;3;3;3	3;3;3;3;3	3;3;3;3;3	IPI00604773;IPI01018674;IPI00299116;IPI00926191;IPI01012180
P49902	NT5C2	2926;3390;8241	2833;3275;7915	3;3;2;2;1;1;1;1	3;3;2;2;1;1;1;1	3;3;2;2;1;1;1;1	IPI00029054;IPI01013382;IPI00902778;IPI00552464;IPI00642843;IPI00552314;IPI01021342;IPI00979827
O95197-3	RTN3	7431;8539	7140;8199	2;1;1;1;1;1;1;1	2;1;1;1;1;1;1;1	2;1;1;1;1;1;1;1	IPI00028946;IPI00743293;IPI00398795;IPI01009279;IPI00412154;IPI00394896;IPI00922617;IPI01010161
P09960-1	LTA4H	3290;7788	3182;7482	2;2;2;2	2;2;2;2	2;2;2;2	IPI00219077;IPI00790203;IPI00514090;IPI00793812
P52948-5	NUP98	2049;6645	1983;6373	2;2;2;2;2;1;1;1	2;2;2;2;2;1;1;1	2;2;2;2;2;1;1;1	IPI00006038;IPI00337397;IPI00216688;IPI00953181;IPI01011651;IPI00337395;IPI00936708;IPI00337396
Q14192	FHL2	2110;4679	2043;4506	2;2;1;1	2;2;1;1	2;2;1;1	IPI00396967;IPI00743342;IPI00398431;IPI00916700
P52209	PGD	925;1328;1354;2825;3264;3934;5901;6236;6237;6574;6765;7215;7524;8201;9676	888;1283;1309;2734;3158;3789;5687;5987;6303;6490;6930;7227;7878;9290	14;14;13;10;8	14;14;13;10;8	14;14;13;10;8	IPI00219525;IPI01012504;IPI01011970;IPI01009353;IPI00910950
P54577	Yars	4190;5931;7107;8684;8727;8728	4034;5716;6826;8336;8378;8379	6	6	6	IPI00007074
P32322	Pycr1	1231;1572;4055;6133;6134;7757	1187;1518;3903;5900;7452	5;5;5;5;2	5;5;5;5;2	4;4;4;4;2	IPI00550882;IPI00941557;IPI00376503;IPI00922289;IPI00260769
P26639	Tars	185;1246;2313;2381;2416;2598;2730;4202;4351;4733;4830;5031;9294;9601;9655	182;1202;2239;2305;2339;2517;2641;4045;4189;4557;4652;4845;8921;9220;9270	15;15;12;12;2;2;2;1;1;1	15;15;12;12;2;2;2;1;1;1	15;15;12;12;2;2;2;1;1;1	IPI00908949;IPI00329633;IPI01012172;IPI00910719;IPI00966149;IPI00967989;IPI00966670;IPI00328082;IPI01014077;IPI00892711
O75874	IDH1	1084;2274;3019;4199;5265;5915;6437;7465;7621;8637	1044;2203;2923;4042;5070;5700;6172;7171;7322;8294	10;4;2;2	10;4;2;2	9;3;2;2	IPI00027223;IPI00925000;IPI00927373;IPI00924576
P45880-1	vdac2	5794;7430;8266;9292;9653;9978	5583;7139;7939;8919;9268;9578	6;6;6;6;6;5;3;3;1	6;6;6;6;6;5;3;3;1	6;6;6;6;6;5;3;3;1	IPI00902560;IPI00216026;IPI00455531;IPI00024145;IPI00917420;IPI01010050;IPI00647902;IPI00647442;IPI00844015
Q9NTJ3-1	SMC4	2482;3037;3887;4213;4559;5234;6409;6412;7241;7356	2404;2941;3744;4055;4390;5042;6145;6148;6955;7066	10;10;10;6;2;1;1	10;10;10;6;2;1;1	10;10;10;6;2;1;1	IPI00411559;IPI00793575;IPI00328298;IPI00947372;IPI00945252;IPI00792419;IPI00947558
Q7Z6Z7-1	HUWE1	2401;3226;4119;5194;7003;7437;7595;8050;8624;9142;9216	2324;3122;3964;5004;6725;7145;7296;7737;8281;8776;8848	11;11;11;8;5;2	11;11;11;8;5;2	10;10;10;8;4;2	IPI00179298;IPI00643153;IPI00445401;IPI00746059;IPI00642197;IPI01011389
Q13185	CBX3	885;2737;3895;4770;9625	851;2647;3752;4593;9243	5;1;1;1;1	5;1;1;1;1	4;1;1;1;1	IPI00297579;IPI00878669;IPI01014449;IPI00917509;IPI00917646
P17844	DDX5	557;2502;2615;3140;4552;5256;6119;6381;6824;7119;8288;8371;8460;8629	536;2424;2533;3039;4383;5061;5887;6119;6548;6838;7961;8040;8124;8286	13;13;12;10	13;13;12;10	9;9;8;6	IPI00017617;IPI00947363;IPI00798375;IPI00984405
Q15427	SF3B4	9383;9384	9009	1	1	1	IPI00017339
Q8WX93-3	palld	932;1410;1465;1831;2177;3782;4691;4985;5284;7407;9177;9393	895;1361;1413;1771;2107;3645;4517;4799;5089;7116;8809;9018	12;12;11;11;11;10;10;9;5;2;1	12;12;11;11;11;10;10;9;5;2;1	12;12;11;11;11;10;10;9;5;2;1	IPI00856116;IPI00855763;IPI00856001;IPI00945254;IPI00383645;IPI00945423;IPI00292009;IPI00856046;IPI00967286;IPI00873173;IPI00965469
P99999	CYCS	144;2819;2820;4754;6095;8280	142;2728;2729;4577;5867;7953	6;5;1	6;5;1	6;5;1	IPI00465315;IPI00917605;CON__P62894
Q14444-1	Caprin1	5521;6904;6921;7529;7943;8654;9971	5318;6626;6643;7232;7631;8311;9571	7;7;6;4;3;3	7;7;6;4;3;3	7;7;6;4;3;3	IPI00783872;IPI00873926;IPI00982178;IPI00978969;IPI00910763;IPI00975644
Q09666	ahnak	173;391;1575;2076;2280;2281;2282;2283;2284;2765;3003;3006;3060;3263;3724;3726;3944;3945;4186;4221;4222;4223;4224;4225;5011;5341;5996;6007;6008;6172;8833;8834;8835;8836;8837;8838;8839;8841;8842;8843;8844;8845;8846;8848;8870;8871;8872;8874;8875;8891;8913;8997;9026;9111;9112;9113;9114;9115;9125;9430	171;379;1521;2010;2209;2210;2211;2212;2674;2908;2911;2961;3157;3589;3591;3798;3799;4030;4063;4064;4065;4066;4825;5144;5778;5787;5788;5933;8480;8481;8482;8483;8484;8485;8486;8488;8489;8490;8491;8492;8493;8495;8516;8517;8518;8520;8521;8536;8558;8638;8667;8746;8747;8748;8749;8750;8760;9054	58;17;17;1;1;1;1;1;1	58;17;17;1;1;1;1;1;1	58;17;17;1;1;1;1;1;1	IPI00021812;IPI00979442;IPI01012911;IPI00031605;IPI00979461;IPI00975545;IPI00981507;IPI00978926;IPI00980977
P61457	Pcbd1	1096;4953	1056;4768	2	2	2	IPI00218568
P32320	cda	2602;7270	2521;6983	2;1	2;1	2;1	IPI00027983;IPI01018243
O95817	bag3	80;1593;3208;3715;7912;7954	80;1539;3105;3580;7602;7641	6;4	6;4	6;4	IPI00641582;IPI00639961
P33240-1	cstf2	59;3211;3212;8026	59;3108;7713	3;3;3;3;2;1	3;3;3;3;2;1	3;3;3;3;2;1	IPI00902991;IPI00013256;IPI00607841;IPI00909622;IPI01012987;IPI00550906
P32754	hpd	2102;3469;6168	2035;3346;5930	3;3;2	3;3;2	3;3;2	IPI00218297;IPI00795913;IPI00913977
P40763-1	Stat3	2335;4056;6188;6901;7663	2261;3904;5946;6623;7363	5;5;5;3	5;5;5;3	5;5;5;3	IPI00784414;IPI00306436;IPI00412752;IPI01013059
Q15637-5	Sf1	3474;3475;4032;6011;8651;9258;9259	3351;3881;5790;8308;8888	5;5;5;5;5;5;3;3;1;1;1	5;5;5;5;5;5;3;3;1;1;1	5;5;5;5;5;5;3;3;1;1;1	IPI00386119;IPI00386114;IPI00294627;IPI00386117;IPI00852591;IPI00386120;IPI00941553;IPI00984197;IPI00386118;IPI00945721;IPI00383251;IPI00917108
P28072	PSMB6	4819;7573	4641;7275	2	2	2	IPI00000811
P38606	Atp6v1a	1694;5562;7463;8274;8996	1637;5359;7169;7947;8637	5;5;2;2	5;5;2;2	5;5;2;2	IPI00007682;IPI00946636;IPI01010714;IPI00946476
Q04323-1	Ubxn1	224;404	220;392	2;2;1;1;1;1	2;2;1;1;1;1	2;2;1;1;1;1	IPI00027378;IPI00982790;IPI00396563;IPI00978573;IPI01014777;IPI00910402
P80723-1	Basp1	25;172;189;592;593;3214;4748;7441	25;170;186;571;572;3110;4571;7149	8;6	8;6	8;6	IPI00299024;IPI00908521
P60903	S100A10	1562;6815;6816	1508;6540	2	2	2	IPI00183695
Q9BS40	LXN	71;2100	71;2033	2	2	2	IPI00106687
Q86TC9-1	Mypn	22;8792	22;8439	2;2;1	2;2;1	2;2;1	IPI00645179;IPI00871200;IPI00477680
P16435	por	3362;8501	3250;8161	2;2;2;2;2;2;2	2;2;2;2;2;2;2	2;2;2;2;2;2;2	IPI00927647;IPI00470467;IPI00903331;IPI00910178;IPI01010631;IPI00908675;IPI00973067
Q12849	Grsf1	2010;2905;8628	1945;2812;8285	3;3;3;3;3;3;2	3;3;3;3;3;3;2	3;3;3;3;3;3;2	IPI00478657;IPI00940450;IPI00965777;IPI00956049;IPI01012720;IPI00979483;IPI00914953
P56270	MAZ	7533	7236	1;1;1;1;1	1;1;1;1;1	1;1;1;1;1	IPI00008800;IPI00168859;IPI01014257;IPI00869267;IPI01009451
P13928	ANXA8	2813;7966	2722;7653	2;2;2;2;2;2;1;1;1;1;1	2;2;2;2;2;2;1;1;1;1;1	2;2;2;2;2;2;1;1;1;1;1	IPI00872295;IPI00883636;IPI00910108;IPI01009431;IPI01014192;IPI00908556;IPI00924896;IPI00914849;IPI00871158;IPI00872852;IPI01015235
Q8NAF0	ZNF579	3236	3131	1	1	1	IPI00167985
Q9UH99	SUN2	976;4041;4184;7233	938;3890;4028;6947	4;4;4;1	4;4;4;1	4;4;4;1	IPI00295940;IPI00878697;IPI00941110;IPI00877626
P50238	crip1	1345;2742;8469;8470	1300;2652;8132	3	3	3	IPI00215611
Q14353	Gamt	9604;9991;10052	9223;9591;9652	3;1;1	3;1;1	3;1;1	IPI00030182;IPI00334683;IPI00909640
P02794	FTHL3	347;1765;4909	337;1708;4725	3;3;3;3;1	3;3;3;3;1	3;3;3;3;1	IPI00554521;IPI01013419;IPI00981633;IPI00978197;IPI00929498
O60306	aqr	3081;6786	2982;6511	2;2	2;2	2;2	IPI00796835;IPI00297572
Q9BUT9-1	fam195a	2519;5657	2438;5449	2;1	2;1	2;1	IPI00042604;IPI00063942
Q5JSH3-1	WDR44	3438;9724	3320;9335	2;2;2;1;1	2;2;2;1;1	2;2;2;1;1	IPI00444371;IPI00872729;IPI00639995;IPI00909478;IPI00807730
P05204	HMGN2	5700;6746;6747	5491;6472;6473	3;2;2;2;2	3;2;2;2;2	3;2;2;2;2	IPI00217950;IPI01008742;IPI00018842;IPI00978575;IPI00171277
Q9BTY7	FAM203A	409;453	397;440	2;2	2;2	2;2	IPI00009335;IPI00741734
Q9NZL9-1	MAT2B	757;1563	731;1509	2;2;2;2;1;1;1;1;1	2;2;2;2;1;1;1;1;1	2;2;2;2;1;1;1;1;1	IPI00002324;IPI00181717;IPI00845510;IPI00845294;IPI00921893;IPI00973068;IPI00974257;IPI00845444;IPI00981635
P29966	MARCKS	1519;2645	1465;2560	2	2	2	IPI00219301
P42330	akr1c3	599;3355;5516;6495;6970;9872	578;3244;5313;6228;6692;9479	4;2;1	4;2;1	4;2;1	IPI00291483;IPI01014057;IPI00909541
Q8TE73	DNAH5	7351;9700	7061;9314	2	2	2	IPI00152653
Q71RC2-4	Larp4	3478;4232;5829	3354;4073;5616	3;3;3;3;2;2;2;2;2;1;1	3;3;3;3;2;2;2;2;2;1;1	3;3;3;3;2;2;2;2;2;1;1	IPI00797384;IPI00395347;IPI00982060;IPI00395837;IPI00955044;IPI00043638;IPI00788748;IPI00418689;IPI01012507;IPI00955019;IPI01021803
P16989-1	CSDAP1	1488;6324;6325;7308;7311;8027;9209;9610	1436;6064;6065;7018;7021;7714;8841;9229	3;2;1;0;0;2;0	3;2;1;0;0;2;0	3;2;1;0;0;2;0	IPI00031801;IPI00219148;IPI00219147;IPI00555698;IPI00250153;IPI01014994;IPI00556023
Q9Y2W2	WBP11	603;6300	582;6042	2;2;1	2;2;1	2;2;1	IPI00170786;IPI00910819;IPI01013891
B4DT67	Spats2l	186;7172	183;6889	2;2;2;2;1;1;1;1;1;1;1;1;1;1;1	2;2;2;2;1;1;1;1;1;1;1;1;1;1;1	2;2;2;2;1;1;1;1;1;1;1;1;1;1;1	IPI00023532;IPI00956176;IPI00916831;IPI00103027;IPI00917040;IPI00926880;IPI00915844;IPI00917531;IPI01013077;IPI00916544;IPI00917948;IPI00917360;IPI00916902;IPI00915976;IPI01010942
Q9UPN3-1	MACF1	322;1437;5036;5837	313;1386;4850;5623	3;3;3;3;3;3;3	3;3;3;3;3;3;3	3;3;3;3;3;3;3	IPI00550385;IPI00432363;IPI00256861;IPI01012199;IPI01018940;IPI00478226;IPI01018834
Q14764	MVP	1108;4125;7516;9398	1068;3970;7221;9023	4;3	4;3	4;3	IPI00000105;IPI00910019
Q9NQX3-2	GPHN	1434;3076	1383;2977	2;2;2;2;2;2	2;2;2;2;2;2	2;2;2;2;2;2	IPI00922682;IPI00184477;IPI00016006;IPI01010764;IPI01025811;IPI01025137
P46013-1	MKI67	7507;8573	7212;8233	2;2;2	2;2;2	2;2;2	IPI00004233;IPI01011955;IPI00413173
P98179	RBM3	527;2709;5112;10054	507;2621;4923;9654	4	4	4	IPI00024320
Q8NFJ5	Gprc5a	815;6582;8503	784;6311;8163	3;1	3;1	3;1	IPI00022624;IPI01011397
Q13228-1	SELENBP1	3187;5540	3084;5337	2;2;2;1	2;2;2;1	2;2;2;1	IPI00012303;IPI00942368;IPI00954180;IPI00852838
Q9H6F5	CCDC86	504;4622	486;4451	2;1	2;1	2;1	IPI00012199;IPI00412260
P53367-1	arfip1	3543;3756	3417;3621	2;2;1;1	2;2;1;1	2;2;1;1	IPI00021258;IPI00216520;IPI00744919;IPI00910325
O75506	HSBP1	8670	8325	1	1	1	IPI00935516
Q9NZ23	YA61	336;9358	326;8984	2	2	2	IPI00386208
Q05932-1	FPGS	2902;7823	2810;7515	2;2;2	2;2;2	2;2;2	IPI00016745;IPI00552800;IPI00759584
Q02978	Slc25a11	93;788	93;760	2;2;2	2;2;2	2;2;2	IPI00219729;IPI00945233;IPI00797802
P30530-1	AXL	5193	5003	1;1	1;1	1;1	IPI00296992;IPI00397361
O75880	sco1	3759;6501	3638;6299	2;2	2;2	2;2	IPI00027233;IPI00793065
Q4G112-1	HSF5	1837;7653	1776;7353	2;1	2;1	2;1	IPI00297953;IPI00983044
Q969G5	PRKCDBP	4491	4323	1;1	1;1	1;1	IPI00976461;IPI00056334
Q13501-1	sqstm1	1070;3654;4840;6552;6724;7154;7968;8734	1030;3521;4662;6281;6450;6873;7655;8385	8;7;6;5;4;4;4;3;2;1;1	8;7;6;5;4;4;4;3;2;1;1	8;7;6;5;4;4;4;3;2;1;1	IPI00179473;IPI00784104;IPI00965064;IPI00879205;IPI00878713;IPI00964222;IPI01013652;IPI00908710;IPI00013676;IPI00964217;IPI00964756
Q58FF2	GRP94c	1544;4349;6005;6006	1490;4187;5786	1	1	1	IPI00556482

Uniprot Acc	Protein name	ENSEMBL	Proteins	Peptides	Razor + unique peptides	Unique peptides	Sequence coverage [%]	Unique + razor sequence coverage [%]	Unique sequence coverage [%]	Mol. weight [kDa]	Sequence length	Sequence lengths	PEP	Ratio H/L	Ratio H/L normalized	Ratio H/L normalized Significance A	Ratio H/L normalized Significance B	Ratio H/L variability [%]	Ratio H/L count	Intensity	Intensity L	Intensity H
O94813-1	Slit homolog 2 protein	ENSEMBL:ENSP00000273739	4	2	2	2	1,6	1,6	1,6	171,34	1542	1542;1529;1525;1521	0,015038	0,06267	0,03912	1,18E-66	8,78E-76	446,84	2	41112000	33878000	7233900
E9PBJ5	Succinate dehydrogenase [ubiquinone] flavoprotein subunit, mitochondrial	ENSEMBL:ENSP00000427703	6	16	1	0	43,3	1,6	0	67,299	616	616;519;258;126;263;125	8,90E-265	0,29425	0,17754	1,39E-20	1,06E-17	68,127	2	5487400	4395200	1092200
Q96QD8-1	solute carrier family 38, member 2	ENSEMBL:ENSP00000256689	3	3	3	3	10,5	10,5	10,5	56,025	506	506;406;88	5,00E-84	0,37491	0,24794	4,48E-14	3,16E-12	14,581	4	11259000	8258900	3000100
P49770	eukaryotic translation initiation factor 2B, subunit 2 beta, 39kDa	ENSEMBL:ENSP00000266126	3	1	1	1	2,6	2,6	2,6	38,989	351	351;206;200	0,011493	0,39526	0,26197	3,95E-13	1,98E-11	7,6106	2	9526900	6625900	2901000
P05161	ISG15 ubiquitin-like modifier	ENSEMBL:ENSP00000368699	1	6	6	6	51,5	51,5	51,5	17,887	165	165	1,28E-79	0,41559	0,26742	8,75E-13	2,36E-14	12,796	16	1,14E+08	81510000	32117000
P48681	nestin	ENSEMBL:ENSP00000357206	2	1	1	1	0,7	0,7	0,7	177,44	1621	1621;594	0,014237	0,47781	0,30772	1,44E-10	1,03E-11	5,34	2	48429000	32897000	15532000
Q9ULE4	family with sequence similarity 184, member B	ENSEMBL:ENSP00000265018	1	1	1	1	1,1	1,1	1,1	121,04	1060	1060	3,44E-05	0,51808	0,34188	4,67E-09	4,52E-10	9,6734	6	54397000	36741000	17657000
P21980-1	transglutaminase 2 (C polypeptide, protein-glutamine-gamma-glutamyltransferase)	ENSEMBL:ENSP00000355330	5	5	5	5	12,2	12,2	12,2	77,328	687	687;627;606;548;349	1,68E-48	0,55454	0,35041	1,01E-08	1,27E-09	9,6142	6	26795000	17795000	8999900
O15143	actin related protein 2/3 complex, subunit 1B, 41kDa; similar to Actin-related protein 2/3 complex subunit 1B (ARP2/3 complex 41 kDa subunit) (p41-ARC)	ENSEMBL:ENSP00000252725;ENSP00000389631	10	2	2	2	7,3	7,3	7,3	40,949	372	372;213;133;130;128;108;102;83;32;21	2,25E-13	0,60053	0,36299	2,96E-08	2,64E-07	81,654	2	7358900	3937900	3421000
Q96RV3-1	pecanex homolog (Drosophila)	ENSEMBL:ENSP00000304192	5	1	1	1	0,7	0,7	0,7	258,67	2341	2341;2269;2230;425;95	0,001863	0,56338	0,36496	3,49E-08	3,03E-07	1,4482	2	9398900	6158600	3240300
P07203	Glutathione peroxidase 1	ENSEMBL:ENSP00000407375	1	2	2	2	17,7	17,7	17,7	22,041	203	203	8,76E-12	0,66243	0,39271	2,89E-07	1,83E-06	99,16	2	8332100	5749000	2583100
P08243	asparagine synthetase	ENSEMBL:ENSP00000175506;ENSP00000377845;ENSP0000037784	8	5	5	5	15,5	15,5	15,5	64,369	561	561;540;478;401;215;162;119;63	9,13E-32	0,63099	0,40958	9,15E-07	2,08E-07	8,2819	5	23938000	14205000	9732700
P00966	argininosuccinate synthetase 1	ENSEMBL:ENSP00000253004;ENSP00000361469;ENSP0000036147 ;ENSP00000361471	5	7	7	7	26,4	26,4	26,4	50,817	450	450;295;198;179;169	4,82E-72	0,71245	0,44889	9,52E-06	2,74E-06	9,4966	17	1,32E+08	81208000	50595000
O43175	phosphoglycerate dehydrogenase	ENSEMBL:ENSP00000358417	3	7	7	7	20,3	20,3	20,3	56,65	533	533;499;406	1,56E-146	0,74906	0,45245	1,15E-05	3,41E-06	10,365	10	62568000	37187000	25381000
P25815	S100 calcium binding protein P	ENSEMBL:ENSP00000296370	1	3	3	3	31,6	31,6	31,6	10,4	95	95	6,70E-28	0,78995	0,47259	3,22E-05	1,10E-05	18,414	9	51051000	28941000	22110000
P23381-1	tryptophanyl-tRNA synthetase	ENSEMBL:ENSP00000347495;ENSP00000376620;ENSP0000045146	28	15	15	15	48,6	48,6	48,6	53,165	471	471;430;185;227;224;212;177;152;150;154;153;140;124;114;107;105;97;105;86;70;68;63;58;55;40;36;33;26	2,73E-180	0,80058	0,47946	4,47E-05	1,59E-05	21,352	23	67909000	39706000	28203000
P09923	alkaline phosphatase, intestinal	ENSEMBL:ENSP00000295463	1	8	5	5	23,5	16,9	16,9	56,811	528	528	7,74E-141	0,79014	0,48003	4,59E-05	1,64E-05	8,0927	12	60404000	35243000	25161000
P32929-1	cystathionase (cystathionine gamma-lyase)	ENSEMBL:ENSP00000359976	3	3	3	3	15,8	15,8	15,8	44,508	405	405;373;361	1,17E-58	0,75364	0,49454	8,89E-05	3,63E-05	11,237	5	18998000	11493000	7504500
Q5H9R7-5	SAPS domain family, member 3	ENSEMBL:ENSP00000377390	12	2	2	2	2,6	2,6	2,6	98,484	879	879;879;873;867;844;838;827;793;791;641;556;112	1,42E-62	0,81506	0,50911	0,000165633	0,000415309	88,829	3	5552200	3460500	2091700
Q9Y617-1	chromosome 8 open reading frame 62; phosphoserine aminotransferase 1	ENSEMBL:ENSP00000365773	3	11	11	11	32,5	32,5	32,5	45,355	415	415;324;194	9,49E-117	0,84324	0,51757	0,000233404	0,000103841	12,824	25	1,32E+08	71296000	61092000
P07951-2	tropomyosin 2 (beta)	ENSEMBL:ENSP00000367542	7	7	3	3	26,8	19	19	32,989	284	284;248;284;284;322;284;303	4,37E-83	1,00817	0,54460	0,000644024	0,00133682	48,116	3	5116300	2617100	2499200
Q969X5-1	endoplasmic reticulum-golgi intermediate compartment (ERGIC) 1	ENSEMBL:ENSP00000377374	7	4	4	4	15,2	15,2	15,2	32,592	290	290;235;183;167;159;138;105	4,97E-16	0,80959	0,54517	0,000656995	0,00136001	12,421	4	5858500	2890100	2968300
Q96C24-1	synaptotagmin-like 4	ENSEMBL:ENSP00000403556	3	1	1	1	1,8	1,8	1,8	76,209	673	673;671;349	0,007832	0,92920	0,55191	0,00083065	0,000436642	6,0316	4	50184000	26692000	23491000
Q14651	plastin 1 (I isoform)	ENSEMBL:ENSP00000336831;ENSP00000387890;ENSP0000041870	2	3	1	1	6,4	3	3	70,253	629	629;266	2,02E-12	0,84710	0,56760	0,00139799	0,00261086	11,379	3	2279300	1330600	948700
P41250	glycyl-tRNA synthetase	ENSEMBL:ENSP00000373918	1	12	12	12	30,4	30,4	30,4	83,165	739	739	1,20E-184	0,88005	0,57084	0,00154997	0,000882805	28,482	34	2,8E+08	148110000	132010000
G5E9P9	RAP1, GTP-GDP dissociation stimulator 1	ENSEMBL:ENSP00000407157	9	2	2	2	5,4	5,4	5,4	66,316	607	607;608;607;559;558;516;148;129;122	0,00165	0,87123	0,58696	0,00253697	0,00437398	90,274	2	1145100	718900	426160
Q01650	solute carrier family 7 (cationic amino acid transporter, y+ system), member 5	ENSEMBL:ENSP00000261622	1	3	3	3	9,3	9,3	9,3	55,01	507	507	2,74E-11	0,90621	0,60742	0,00452765	0,00296036	12,608	3	14328000	6406100	7921500
P14324	farnesyl diphosphate synthase (farnesyl pyrophosphate synthetase, dimethylallyltranstransferase, geranyltranstransferase)	ENSEMBL:ENSP00000349078;ENSP00000357340	5	6	6	6	21,7	21,7	21,7	48,275	419	419;353;109;348;357	1,21E-49	0,92251	0,60868	0,00468415	0,00307462	10,103	9	47219000	25430000	21789000
P07602-2	prosaposin	ENSEMBL:ENSP00000362212;ENSP00000378392	5	2	2	2	5	5	5	61,692	559	559;527;527;524;450	7,24E-39	0,92635	0,62035	0,00636735	0,0043253	6,6078	9	66529000	35288000	31241000
Q9GZM8-2	nudE nuclear distribution gene E homolog (A. nidulans)-like 1	ENSEMBL:ENSP00000384963	6	2	2	2	7	7	7	44,448	400	400;367;345;328;346;335	2,41E-21	1,02556	0,62093	0,00646239	0,00439991	73,258	2	26858000	15189000	11670000
P11766	alcohol dehydrogenase 5 (class III), chi polypeptide, pseudogene 4; alcohol dehydrogenase 5 (class III), chi polypeptide	ENSEMBL:ENSP00000296412	5	2	2	2	10,2	10,2	10,2	39,724	374	374;262;115;95;56	9,26E-17	0,99930	0,62116	0,0065079	0,00990641	68,068	2	2511600	1080500	1431100
P06737	phosphorylase, glycogen, liver	ENSEMBL:ENSP00000216392	3	4	4	4	8,6	8,6	8,6	97,147	847	847;819;813	1,14E-32	1,04341	0,62551	0,00725918	0,0109059	13,744	4	10390000	5106000	5283600
P30740	serpin peptidase inhibitor, clade B (ovalbumin), member 1	ENSEMBL:ENSP00000370115	3	4	4	4	15,3	15,3	15,3	42,741	379	379;341;228	3,51E-74	1,03836	0,62952	0,00802327	0,00560459	12,809	4	62157000	31060000	31098000
Q96EE3-1	SEH1-like (S. cerevisiae)	ENSEMBL:ENSP00000382779	2	2	2	2	5,7	5,7	5,7	46,578	421	421;360	1,76E-06	1,06910	0,63975	0,0102758	0,0147678	22,882	2	3177600	1096600	2081000
Q16539-1	mitogen-activated protein kinase 14	ENSEMBL:ENSP00000229794	6	2	2	2	8,3	8,3	8,3	41,293	360	360;360;283;307;297;133	9,52E-08	1,01963	0,64144	0,0106919	0,0152888	25,532	2	7837000	4112300	3724700
P08195-4	solute carrier family 3 (activators of dibasic and neutral amino acid transport), member 2	ENSEMBL:ENSP00000367124;ENSP00000440001	12	20	20	20	39,8	39,8	39,8	71,122	661	661;631;630;599;568;529;511;145;135;153;122;47	0	1,06341	0,64379	0,0112973	0,00822043	24,042	45	3,61E+08	179790000	180890000
P05413	fatty acid binding protein 3, muscle and heart (mammary-derived growth inhibitor)	ENSEMBL:ENSP00000362817	1	2	2	2	17,3	17,3	17,3	14,858	133	133	1,75E-10	1,00781	0,64445	0,011473	0,0162601	6,4233	3	4581400	2228700	2352800
Q13907-2	isopentenyl-diphosphate delta isomerase 1	ENSEMBL:ENSP00000370748	2	2	2	2	12,3	12,3	12,3	32,485	284	284;227	2,11E-18	0,98338	0,64574	0,0118209	0,00860962	4,1307	4	13263000	6689700	6572900
Q9UH65	SWAP switching B-cell complex 70kDa subunit	ENSEMBL:ENSP00000315630	3	2	2	2	4,4	4,4	4,4	68,997	585	585;527;149	7,75E-07	1,00558	0,64800	0,0124511	0,0174655	47,495	2	2151600	1292100	859590
Q7L1Q6	basic leucine zipper and W2 domains 1 pseudogene 1; basic leucine zipper and W2 domains 1 like 1; basic leucine zipper and W2 domains 1	ENSEMBL:ENSP00000394316	9	2	2	2	7,3	7,3	7,3	51,281	451	451;423;419;353;216;162;155;135;116	4,02E-16	1,02453	0,64906	0,0127536	0,0178359	48,106	2	2272700	1290100	982560
Q16719	kynureninase (L-kynurenine hydrolase)	ENSEMBL:ENSP00000264170;ENSP00000386731	4	14	14	14	38,9	38,9	38,9	52,351	465	465;307;148;76	1,45E-88	1,05554	0,64969	0,0129384	0,0095663	11,977	16	56902000	28701000	28201000
Q14139-2	ubiquitination factor E4A (UFD2 homolog, yeast)	ENSEMBL:ENSP00000387362	3	1	1	1	1,5	1,5	1,5	123,52	1073	1073;1066;392	0,003316	1,08058	0,65407	0,0142782	0,0196877	19,225	2	3506400	1539100	1967300
P42785	prolylcarboxypeptidase (angiotensinase C)	ENSEMBL:ENSP00000377055	10	2	2	2	5,8	5,8	5,8	58,099	517	517;496;391;192;183;148;145;164;116;100	8,68E-17	1,03208	0,66011	0,016312	0,0221223	2,9449	2	5737000	3027100	2709900
Q14116	interleukin 18 (interferon-gamma-inducing factor)	ENSEMBL:ENSP00000280357;ENSP00000434161	2	2	2	2	25,4	25,4	25,4	22,326	193	193;189	8,43E-29	1,08601	0,66503	0,0181338	0,024273	10,889	2	5498800	2820400	2678400
O60568	procollagen-lysine, 2-oxoglutarate 5-dioxygenase 3	ENSEMBL:ENSP00000223127	2	5	5	5	10,7	10,7	10,7	84,784	738	738;264	1,54E-19	1,04679	0,66993	0,0201093	0,0155176	13,561	6	23955000	11542000	12413000
Q8NBJ5	glycosyltransferase 25 domain containing 1	ENSEMBL:ENSP00000252599	2	4	4	4	8,8	8,8	8,8	71,635	622	622;233	6,27E-20	1,21359	0,67060	0,0203939	0,015761	63,591	4	32676000	13534000	19142000
P30533	low density lipoprotein receptor-related protein associated protein 1	ENSEMBL:ENSP00000421922	1	2	2	2	8,1	8,1	8,1	41,465	357	357	1,83E-19	1,10456	0,67467	0,0221831	0,0289679	15,458	2	5766100	2802800	2963200
O00592-1	podocalyxin-like	ENSEMBL:ENSP00000440518	5	3	3	3	7,3	7,3	7,3	58,875	560	560;558;528;526;516	1,81E-27	1,10833	0,68399	0,0267512	0,0341486	51,674	2	3440600	1894700	1545900
P49902	5'-nucleotidase, cytosolic II	ENSEMBL:ENSP00000339479;ENSP00000383960	8	3	3	3	8,6	8,6	8,6	64,969	561	561;532;408;147;265;199;157;87	7,01E-14	1,07320	0,69290	0,0317825	0,0397412	36,469	3	9230600	5107900	4122800
O95197-3	reticulon 3	ENSEMBL:ENSP00000440874	8	2	2	2	11	11	11	25,609	236	236;1032;1013;920;255;241;214;123	9,09E-26	1,14277	0,69784	0,0348692	0,0285146	11,041	4	28628000	13733000	14895000
P09960-1	leukotriene A4 hydrolase	ENSEMBL:ENSP00000228740	4	2	2	2	6,2	6,2	6,2	69,284	611	611;587;532;508	4,49E-21	1,07876	0,69857	0,035346	0,0289451	2,2936	2	22586000	11342000	11244000
P52948-5	nucleoporin 98kDa	ENSEMBL:ENSP00000316032	8	2	2	2	1,4	1,4	1,4	197,58	1817	1817;1800;1726;1714;1638;937;920;666	1,95E-06	1,14211	0,69881	0,0355063	0,0438169	35,362	2	5955500	3422100	2533400
Q14192	four and a half LIM domains 2	ENSEMBL:ENSP00000344266	4	2	2	2	6,2	6,2	6,2	44,205	389	389;279;151;19	2,56E-23	1,07244	0,70244	0,0379588	0,0313141	7,0119	3	38285000	20366000	17918000
P52209	phosphogluconate dehydrogenase	ENSEMBL:ENSP00000270776	5	14	14	14	41,8	41,8	41,8	53,139	483	483;470;461;429;289	2,74E-254	1,15867	0,70646	0,0408231	0,0343767	10,131	29	2,36E+08	114440000	121960000
P54577	tyrosyl-tRNA synthetase	ENSEMBL:ENSP00000362576	1	6	6	6	15,3	15,3	15,3	59,143	528	528	4,27E-70	1,13157	0,71190	0,0449539	0,0377308	12,455	10	47765000	24384000	23382000
P32322	pyrroline-5-carboxylate reductase 1	ENSEMBL:ENSP00000384949	5	5	5	4	24,3	24,3	20,5	35,98	346	346;319;316;240;272	4,28E-110	1,13440	0,71393	0,0465794	0,0397854	10,973	9	64775000	31203000	33572000
P26639	threonyl-tRNA synthetase	ENSEMBL:ENSP00000387710	10	15	15	15	25,5	25,5	25,5	86,86	756	756;723;619;602;131;129;118;802;766;707	2,39E-110	1,09176	0,71726	0,0493369	0,0424001	22,543	26	1,89E+08	94013000	95103000
O75874	isocitrate dehydrogenase 1 (NADP+), soluble	ENSEMBL:ENSP00000260985;ENSP00000390265;ENSP0000041051	4	10	10	9	30,7	30,7	28,5	46,659	414	414;157;74;62	3,89E-99	1,18703	0,71813	0,0500824	0,0431096	14,137	15	1,02E+08	50302000	52034000
P45880-1	voltage-dependent anion channel 2	ENSEMBL:ENSP00000361635	9	6	6	6	35,3	35,3	35,3	33,371	309	309;294;293;283;282;255;204;195;109	7,68E-108	1,19511	0,72601	0,0571737	0,0499063	12,162	14	1,01E+08	48825000	52487000
Q9NTJ3-1	structural maintenance of chromosomes 4	ENSEMBL:ENSP00000341382;ENSP00000349961	7	10	10	10	12	12	12	147,18	1288	1288;1263;1230;423;139;157;119	5,19E-147	2,29553	1,40964	0,107975	0,0495305	24,489	13	65319000	16482000	48837000
Q7Z6Z7-1	HECT, UBA and WWE domain containing 1	ENSEMBL:ENSP00000262854;ENSP00000340648	6	11	11	10	5,7	5,7	5,3	482,14	4376	4376;4365;4358;3408;1672;1197	1,70E-108	2,29331	1,41741	0,101698	0,0457221	24,811	14	83042000	26448000	56594000
Q13185	similar to chromobox homolog 3; chromobox homolog 3 (HP1 gamma homolog, Drosophila)	ENSEMBL:ENSP00000336687;ENSP00000379670	5	5	5	4	45,9	45,9	39,3	20,811	183	183;189;168;101;62	2,08E-46	2,26937	1,42729	0,0941933	0,0412697	16,672	7	60366000	19054000	41312000
P17844	DEAD (Asp-Glu-Ala-Asp) box polypeptide 5	ENSEMBL:ENSP00000440276	4	14	13	9	28,8	27	19,5	69,147	614	614;603;544;457	2,63E-127	2,39975	1,43258	0,0903745	0,0390481	25,603	21	1,06E+08	35594000	70762000
Q15427	splicing factor 3b, subunit 4, 49kDa	ENSEMBL:ENSP00000271628	1	1	1	1	5,4	5,4	5,4	44,385	424	424	1,72E-26	2,21175	1,45866	0,0735174	0,0296243	4,0697	7	95969000	33530000	62439000
Q8WX93-3	palladin, cytoskeletal associated protein	ENSEMBL:ENSP00000336735	11	12	12	12	12,6	12,6	12,6	150,56	1383	1383;1001;1159;777;672;1123;1106;385;160;164;90	1,76E-147	2,51509	1,47367	0,0651592	0,0252035	18,735	15	76326000	22912000	53414000
P99999	cytochrome c, somatic	ENSEMBL:ENSP00000307786;ENSP00000386270;ENSP0000038727	3	6	6	6	62,9	62,9	62,9	11,749	105	105;101;105	3,76E-102	2,44236	1,49049	0,0568206	0,0209785	13,139	15	2,09E+08	63816000	145060000
Q14444-1	cell cycle associated protein 1	ENSEMBL:ENSP00000340329;ENSP00000434150	6	7	7	7	18,3	18,3	18,3	78,365	709	709;694;628;186;372;178	3,76E-86	2,22608	1,49309	0,0556209	0,020387	19,999	19	1,06E+08	34407000	71930000
Q09666	AHNAK nucleoprotein	ENSEMBL:ENSP00000367263	9	58	58	58	24,5	24,5	24,5	629,09	5890	5890;1714;791;150;144;131;123;121;86	0	2,38874	1,50011	0,0525053	0,0188708	25,036	61	2,74E+08	89674000	184540000
P61457	pterin-4 alpha-carbinolamine dehydratase/dimerization cofactor of hepatocyte nuclear factor 1 alpha	ENSEMBL:ENSP00000299299	1	2	2	2	27,9	27,9	27,9	11,999	104	104	1,70E-57	2,34626	1,51902	0,0448814	0,0152898	3,5807	2	52115000	15504000	36611000
P32320	cytidine deaminase	ENSEMBL:ENSP00000364212	2	2	2	2	26,7	26,7	26,7	16,185	146	146;82	4,66E-86	2,98054	1,55104	0,0342621	0,0106418	16,729	8	1,15E+08	33395000	81348000
O95817	BCL2-associated athanogene 3	ENSEMBL:ENSP00000358081	2	6	6	6	22,8	22,8	22,8	61,594	575	575;325	8,72E-43	2,93436	1,57851	0,0270656	0,0451056	19,275	6	40178000	9331500	30846000
P33240-1	cleavage stimulation factor, 3' pre-RNA, subunit 2, 64kDa	ENSEMBL:ENSP00000387996	6	3	3	3	12,4	12,4	12,4	62,928	597	597;577;560;553;438;616	2,31E-14	2,51042	1,57923	0,0268969	0,0448761	8,7553	7	26869000	7161400	19707000
P32754	4-hydroxyphenylpyruvate dioxygenase	ENSEMBL:ENSP00000289004	3	3	3	3	14,2	14,2	14,2	44,934	393	393;354;377	3,25E-67	2,37468	1,58323	0,0259808	0,007338	13,834	7	58023000	18284000	39739000
P40763-1	signal transducer and activator of transcription 3 (acute-phase response factor)	ENSEMBL:ENSP00000264657	4	5	5	5	11,9	11,9	11,9	88,067	770	770;769;722;672	1,40E-141	2,45339	1,58765	0,0250018	0,0422802	59,233	9	29123000	9640700	19482000
Q15637-5	splicing factor 1	ENSEMBL:ENSP00000366604	12	5	5	5	14,5	14,5	14,5	80,62	764	764;639;638;623;613;571;548;452;433;292;110;62	1,42E-83	2,61657	1,59424	0,0236093	0,0064521	22,413	15	75306000	18005000	57301000
P28072	proteasome (prosome, macropain) subunit, beta type, 6	ENSEMBL:ENSP00000270586	1	2	2	2	20,9	20,9	20,9	25,357	239	239	6,13E-47	2,40604	1,59551	0,0233487	0,00635654	11,126	7	59952000	21448000	38504000
P38606	ATPase, H+ transporting, lysosomal 70kDa, V1 subunit A	ENSEMBL:ENSP00000273398	4	5	5	5	15,6	15,6	15,6	68,303	617	617;584;334;221	1,67E-74	2,45026	1,60231	0,0220008	0,0380967	31,545	7	26497000	7078200	19419000
Q04323-1	UBX domain protein 1	ENSEMBL:ENSP00000303991	6	2	2	2	22,2	22,2	22,2	33,325	297	297;293;312;290;200;136	2,09E-06	2,73538	1,60839	0,0208576	0,0364769	13,122	2	22030000	6270000	15760000
P80723-1	brain abundant, membrane attached signal protein 1	ENSEMBL:ENSP00000319281	2	8	8	8	60,4	60,4	60,4	22,693	227	227;173	4,35E-91	2,85217	1,62742	0,0176319	0,00435703	21,088	15	1,38E+08	42149000	96288000
P60903	S100 calcium binding protein A10	ENSEMBL:ENSP00000357799;ENSP00000357801	1	2	2	2	35,1	35,1	35,1	11,203	97	97	2,02E-42	2,50470	1,62774	0,0175822	0,00434053	8,8411	12	85081000	25425000	59657000
Q9BS40	latexin	ENSEMBL:ENSP00000264265	1	2	2	2	12,2	12,2	12,2	25,75	222	222	1,72E-35	2,48268	1,62864	0,0174422	0,0493007	18,397	3	3498400	1004200	2494200
Q86TC9-1	myopalladin	ENSEMBL:ENSP00000351790	3	2	2	2	3,3	3,3	3,3	145,26	1320	1320;1320;1045	1,48E-22	2,42954	1,63851	0,0159755	0,046155	26,627	5	5423600	1596400	3827200
P16435	P450 (cytochrome) oxidoreductase	ENSEMBL:ENSP00000390540	7	2	2	2	6,8	6,8	6,8	77,02	680	680;677;633;573;488;452;418	2,73E-33	2,87274	1,64623	0,0149116	0,0277672	30,397	3	32571000	10206000	22365000
Q12849	G-rich RNA sequence binding factor 1	ENSEMBL:ENSP00000254799	7	3	3	3	16	16	16	53,126	480	480;459;453;448;415;393;318	6,75E-22	2,97610	1,65483	0,013804	0,0260808	31,006	6	31577000	8040800	23536000
P56270	MYC-associated zinc finger protein (purine-binding transcription factor)	ENSEMBL:ENSP00000313362	5	1	1	1	2,6	2,6	2,6	51,066	497	497;493;454;253;252	0,000328	2,89126	1,65684	0,0135579	0,0408077	5,4046	2	2343500	674070	1669500
P13928	annexin A8	ENSEMBL:ENSP00000341674	11	2	2	2	7,9	7,9	7,9	43,855	393	393;327;270;141;141;121;365;327;276;276;141	0,000145	2,53177	1,66514	0,0125807	0,0385806	19,427	2	8242100	2501500	5740600
Q8NAF0	zinc finger protein 579	ENSEMBL:ENSP00000320188	1	1	1	1	2,8	2,8	2,8	60,509	562	562	2,52E-06	2,69121	1,68728	0,0102926	0,0331881	8,6685	2	2012400	592980	1419400
Q9UH99	unc-84 homolog B (C. elegans)	ENSEMBL:ENSP00000216064;ENSP00000383992;ENSP0000038574	4	4	4	4	8,5	8,5	8,5	83,964	752	752;738;706;227	1,81E-53	2,91011	1,68776	0,0102473	0,0204841	25,623	4	12622000	3383900	9238400
P50238	cysteine-rich protein 1 (intestinal)	ENSEMBL:ENSP00000332449;ENSP00000376315;ENSP0000038634	1	3	3	3	58,4	58,4	58,4	8,5328	77	77	1,86E-20	2,71791	1,68816	0,0102102	0,00208836	21,574	6	64676000	20662000	44014000
Q14353	guanidinoacetate N-methyltransferase	ENSEMBL:ENSP00000252288	3	3	3	3	22	22	22	26,318	236	236;269;121	1,00E-27	2,53222	1,68819	0,0102075	0,0329821	21,164	4	5969100	1775700	4193400
P02794	ferritin, heavy polypeptide 1; ferritin, heavy polypeptide-like 16; similar to ferritin, heavy polypeptide 1; ferritin, heavy polypeptide-like 3 pseudogene	ENSEMBL:ENSP00000273550	5	3	3	3	20,8	20,8	20,8	21,225	183	183;183;153;113;126	6,27E-27	3,04053	1,69251	0,00981364	0,0197791	29,538	4	19562000	4963300	14599000
O60306	aquarius homolog (mouse)	ENSEMBL:ENSP00000445700	2	2	2	2	3	3	3	171,29	1485	1485;1353	3,15E-08	2,78211	1,69918	0,00923303	0,0305918	4,642	3	7320300	1936400	5384000
Q9BUT9-1	chromosome 16 open reading frame 14	ENSEMBL:ENSP00000305138	2	2	2	2	31,9	31,9	31,9	17,828	160	160;194	3,97E-34	3,05493	1,70175	0,00901798	0,030056	18,236	3	3687300	1054700	2632700
Q5JSH3-1	WD repeat domain 44	ENSEMBL:ENSP00000254029	5	2	2	2	2,5	2,5	2,5	101,37	913	913;905;806;824;429	2,22E-15	2,74952	1,70326	0,00889427	0,0297464	35,522	2	6385200	1581900	4803300
P05204	hypothetical LOC729505; similar to hCG2040565; high-mobility group nucleosomal binding domain 2; similar to high-mobility group nucleosomal binding domain 2	ENSEMBL:ENSP00000355228	5	3	3	3	16,7	16,7	16,7	9,3926	90	90;130;99;95;77	1,55E-14	3,05241	1,70675	0,00861416	0,0177984	10,317	6	22557000	5856000	16701000
Q9BTY7	Protein FAM203A	ENSEMBL:ENSP00000321320	2	2	2	2	11,5	11,5	11,5	42,129	390	390;390	9,07E-14	2,89847	1,72491	0,00728692	0,025618	1,8059	2	3581500	991010	2590500
Q9NZL9-1	methionine adenosyltransferase II, beta	ENSEMBL:ENSP00000325425	9	2	2	2	8,4	8,4	8,4	37,551	334	334;323;306;259;228;198;117;83;82	4,20E-12	2,90554	1,72957	0,00697984	0,0248046	29,794	3	11512000	3876800	7634800
P29966	myristoylated alanine-rich protein kinase C substrate	ENSEMBL:ENSP00000357624	1	2	2	2	15,4	15,4	15,4	31,554	332	332	9,13E-27	2,82829	1,73088	0,00689521	0,0245789	25,526	2	6946800	2696300	4250500
P42330	aldo-keto reductase family 1, member C3 (3-alpha hydroxysteroid dehydrogenase, type II)	ENSEMBL:ENSP00000369927	3	6	4	4	37,8	28,2	28,2	36,853	323	323;204;138	2,39E-182	2,88942	1,74417	0,00609509	0,00104215	24,923	6	1,05E+08	33022000	72088000
Q8TE73	dynein, axonemal, heavy chain 5	ENSEMBL:ENSP00000265104	1	2	2	2	0,7	0,7	0,7	529,01	4624	4624	0,000903	2,85641	1,75420	0,00555065	0,0208919	44,626	2	3261900	694350	2567500
Q71RC2-4	La ribonucleoprotein domain family, member 4	ENSEMBL:ENSP00000415464	11	3	3	3	5,6	5,6	5,6	81,243	730	730;724;723;653;653;605;582;445;375;188;125	2,15E-28	2,64529	1,75654	0,00543051	0,0122595	11,613	3	14149000	3998100	10151000
P16989-1	cold shock domain protein A; cold shock domain protein A pseudogene 1	ENSEMBL:ENSP00000228251	7	8	3	3	20,2	7,8	7,8	40,089	372	372;342;303;134;364;170;268	7,83E-157	3,34526	1,75960	0,00527731	0,0119797	25,27	4	33098000	9276300	23822000
Q9Y2W2	WW domain binding protein 11	ENSEMBL:ENSP00000261167	3	2	2	2	7,8	7,8	7,8	69,997	641	641;607;194	3,19E-08	2,76205	1,80822	0,00333649	0,0142701	52,769	2	3900100	654100	3246000
B4DT67	spermatogenesis associated, serine-rich 2-like	ENSEMBL:ENSP00000387333	15	2	2	2	3,4	3,4	3,4	65,151	588	588;566;558;489;498;266;193;188;173;154;140;115;94;23;13	1,06E-09	3,05829	1,81818	0,00303475	0,0132925	67,367	2	7194300	1885100	5309200
Q9UPN3-1	microtubule-actin crosslinking factor 1	ENSEMBL:ENSP00000362006	7	4	3	3	0,9	0,8	0,8	838,3	7388	7388;5938;5430;5430;5374;5300;5300	2,08E-10	2,70168	1,83661	0,00254471	0,0116508	27,34	4	9148200	2927100	6221100
Q14764	major vault protein	ENSEMBL:ENSP00000349977;ENSP00000378760	2	4	4	4	8,5	8,5	8,5	99,326	893	893;790	1,01E-43	2,96947	1,83773	0,0025177	0,00660027	17,386	8	36261000	9686900	26574000
Q9NQX3-2	Gephyrin	ENSEMBL:ENSP00000438404	6	2	2	2	5,8	5,8	5,8	84,733	782	782;769;736;705;380;280	9,84E-21	2,99312	1,84594	0,00232704	0,0108966	8,7006	2	2647500	724390	1923100
P46013-1	antigen identified by monoclonal antibody Ki-67	ENSEMBL:ENSP00000357643	3	2	2	2	1	1	1	358,69	3256	3256;3255;2896	1,37E-37	2,83399	1,85161	0,00220356	0,0104609	89,7	3	5831300	1988400	3842900
P98179	RNA binding motif (RNP1, RRM) protein 3	ENSEMBL:ENSP00000365946;ENSP00000365950	1	4	4	4	47,8	47,8	47,8	17,17	157	157	2,50E-39	3,10308	1,93633	0,000967354	8,70E-05	9,4663	10	3,66E+08	108400000	257240000
Q8NFJ5	G protein-coupled receptor, family C, group 5, member A	ENSEMBL:ENSP00000014914	2	3	3	3	18,5	18,5	18,5	40,251	357	357;274	1,36E-18	3,13048	1,95450	0,000809177	0,00494416	16,424	3	5904500	1635400	4269100
Q13228-1	selenium binding protein 1	ENSEMBL:ENSP00000357861	4	2	2	2	9,3	9,3	9,3	52,39	472	472;410;408;306	1,67E-24	3,21937	2,01110	0,00046206	0,00169565	2,3476	3	16560000	4027500	12532000
Q9H6F5	coiled-coil domain containing 86	ENSEMBL:ENSP00000227520	2	2	2	2	9,7	9,7	9,7	40,235	360	360;163	1,83E-19	3,64365	2,07379	0,000246881	0,0020355	34,67	2	8807900	2250100	6557800
P53367-1	ADP-ribosylation factor interacting protein 1	ENSEMBL:ENSP00000296557;ENSP00000395083	4	2	2	2	9,4	9,4	9,4	41,738	373	373;341;245;193	1,80E-06	3,28991	2,18996	7,62E-05	0,000845577	29,874	2	3613100	837640	2775500
O75506	Heat shock factor-binding protein 1	ENSEMBL:ENSP00000392896	1	1	1	1	25	25	25	8,5435	76	76	3,49E-26	3,34885	2,21219	6,07E-05	0,000713822	2,1002	2	3691800	968330	2723500
Q9NZ23	Gastric-associated differentially-expressed protein YA61P		1	2	2	2	33,1	33,1	33,1	14,867	136	136	4,58E-36	3,47150	2,23354	4,88E-05	0,000282713	11,971	4	24158000	5289100	18869000
Q05932-1	folylpolyglutamate synthase	ENSEMBL:ENSP00000362344	3	2	2	2	5,5	5,5	5,5	64,608	587	587;561;545	2,36E-10	3,75220	2,26896	3,40E-05	0,000462545	11,475	2	4772700	920920	3851700
Q02978	solute carrier family 25 (mitochondrial carrier; oxoglutarate carrier), member 11	ENSEMBL:ENSP00000225665	3	2	2	2	10,2	10,2	10,2	34,061	314	314;303;263	5,53E-05	3,51346	2,43712	6,01E-06	0,000126765	80,258	2	4994700	1208700	3786000
P30530-1	AXL receptor tyrosine kinase	ENSEMBL:ENSP00000301178	2	1	1	1	1,5	1,5	1,5	98,335	894	894;885	0,000852	4,05022	2,54816	1,90E-06	5,37E-05	13,962	2	2577700	536860	2040800
O75880	SCO cytochrome oxidase deficient homolog 1 (yeast)	ENSEMBL:ENSP00000255390	2	2	2	2	10	10	10	33,814	301	301;270	0,000732	1,71770	2,71540	6,52E-08	6,37E-08	18,142	2	4651400	1705700	2945700
Q4G112-1	heat shock transcription factor family member 5	ENSEMBL:ENSP00000313243	2	2	2	2	11,1	11,1	11,1	65,277	596	596;475	0,015481	4,07614	2,85804	7,63E-08	1,70E-06	243,56	2	20122000	10642000	9480600
Q969G5	Protein kinase C delta-binding protein	ENSEMBL:ENSP00000432047	2	1	1	1	5,1	5,1	5,1	31,088	293	293;261	0,001992	5,42652	2,99383	1,88E-08	1,71E-06	4,6143	2	2740000	499160	2240800
Q13501-1	sequestosome 1	ENSEMBL:ENSP00000374455	11	8	8	8	33,2	33,2	33,2	47,687	440	440;356;378;814;232;190;140;296;420;73;69	3,29E-140	6,34437	3,47911	1,33E-10	4,60E-14	32,225	12	97164000	14532000	82632000
Q58FF2	Heat shock protein 94c		1	3	1	1	7,5	3,3	3,3	51,725	453	453	7,14E-16	46,16805	28,19920	3,51E-69	4,52E-93	117,63	9	1,54E+08	2112000	151810000
