# Supplementary material for: Proteomic analysis of effects by x-rays and heavy ion in HeLa cells
Source: Radiol Oncol. 2014 Apr 25;48(2):142–54. doi: 10.2478/raon-2013-0087 (PMC4078033; doi:10.2478/raon-2013-0087)
Supplement: Supplementary file 2 [file raon-2014-issue2-0142_supp2.pdf]

2 Gy X-ray 1 h SILAC LC-MS/MS

Uniprot Acc	Gene Synonyms	Mod. peptide IDs	Peptide IDs	Peptide counts (all)	Peptide counts (razor+unique)	Peptide counts (unique)	Protein Ids	Protein name	ENSEMBL
Q9H2U1-1	DHX36	1060	1029	1;1;1;1	1;1;1;1	1;1;1;1	IPI00027415;IPI00784170;IPI00922725;IPI00217760	DEAH (Asp-Glu-Ala-His) box polypeptide 36	ENSEMBL:ENSP00000417078
Q92542-2	NCSTN	6532;7242	6327;6969	2;1;1;1;1;1;1	2;1;1;1;1;1;1	2;1;1;1;1;1;1	IPI00375688;IPI00021983;IPI00515093;IPI01010317;IPI00514877;IPI00514793;IPI00513841	nicastrin	ENSEMBL:ENSP00000357042;ENSP00000376047
P05161	ISG15	4244;5305;6307;10127	4104;5133;6110;9755	4	4	4	IPI00375631	ISG15 ubiquitin-like modifier	ENSEMBL:ENSP00000368699
P09913	Ifit2	406;5832	394;5646	2	2	2	IPI00018298	interferon-induced protein with tetratricopeptide repeats 2	ENSEMBL:ENSP00000360891
Q9ULE4	Fam184b	4547	4397	1	1	1	IPI00297208	family with sequence similarity 184, member B	ENSEMBL:ENSP00000265018
Q93096	ptp4a1	673	652	1	1	1	IPI00020164	protein tyrosine phosphatase type IVA, member 1	ENSEMBL:ENSP00000359685
O75880	sco1	3759;6501	3638;6299	2;2	2;2	2;2	IPI00027233;IPI00793065	SCO cytochrome oxidase deficient homolog 1 (yeast)	ENSEMBL:ENSP00000255390
P07203	GPX1	1623;7842	1575;7555	2	2	2	IPI00927606	Glutathione peroxidase 1	ENSEMBL:ENSP00000407375
Q15392	DHCR24	8297	7991	1;1	1;1	1;1	IPI00016703;IPI01008932	24-dehydrocholesterol reductase	ENSEMBL:ENSP00000360316
Q99959-1	Pkp2	8574;8664	8256;8343	2;2;2	2;2;2	2;2;2	IPI00005264;IPI00219688;IPI01013025	plakophilin 2	ENSEMBL:ENSP00000070846
P07951-2	tpm2	816;1756;4567;5042;5177;6367;6564;7891	791;1705;4416;4876;5009;6169;6357;7602	8;8;7;7;7;7;6;3	3;3;2;2;2;2;2;1	2;2;1;1;1;2;1;0	IPI00220709;IPI00646748;IPI00513698;CON__Q3SX28;IPI00013991;IPI00218820;IPI00910712;IPI00909308	tropomyosin 2 (beta)	ENSEMBL:ENSP00000367542
P17096-3 Tax_Id=9606 Gene_Symbol=HMGA1 Isoform HMG-R of High m	HMGA1	5102;8651	4935;8330	2;2;2;1;1;1;1	2;2;2;1;1;1;1	2;2;2;1;1;1;1	IPI00744851;IPI00746310;IPI00179700;IPI00980114;IPI00974030;IPI00450855;IPI00177716	hypothetical LOC100130009; high mobility group AT-hook 1	ENSEMBL:ENSP00000378452
O94888	UBXN7	54;1130;6318	54;1096;6121	3;1	3;1	3;1	IPI00742124;IPI00926703	UBX domain protein 7	ENSEMBL:ENSP00000296328
P21980-1	tgm2	534;6919;9382;10710	518;6664;9037;10316	4;4;4;1	4;4;4;1	4;4;4;1	IPI00294578;IPI01013011;IPI01013460;IPI00218251	transglutaminase 2 (C polypeptide, protein-glutamine-gamma-glutamyltransferase)	ENSEMBL:ENSP00000355330
O75044	srgap2	3721;10165	3602;9792	2;2;2;1;1	2;2;2;1;1	2;2;2;1;1	IPI00479125;IPI00941205;IPI00647466;IPI00376259;IPI00414827	SLIT-ROBO Rho GTPase activating protein 2	ENSEMBL:ENSP00000295713;ENSP00000390898
P11802	Cdk4	550;1400;5210	533;1357;5042	3;2;2;2;2;1;1;1;1	2;1;1;1;1;1;1;1;1	2;1;1;1;1;1;1;1;1	IPI00007811;IPI01021794;IPI01021327;IPI01011274;IPI01022249;IPI01022897;IPI00059926;IPI01022046;IPI01021473	cyclin-dependent kinase 4	ENSEMBL:ENSP00000257904
P61254	RPL26	3683	3567	1;1	1;1	1;1	IPI00433834;IPI00027270	ribosomal protein L26 pseudogene 33; ribosomal protein L26; ribosomal protein L26 pseudogene 16; ribosomal protein L26 pseudogene 19; ribosomal protein L26 pseudogene 6	ENSEMBL:ENSP00000293842
Q6FI13	HIST2H2AA3	323;3827;4898;5133;9747;10258	313;3702;4736;4966;9387;9882	6;5	1;1	1;1	IPI00216457;IPI00339274	histone cluster 2, H2aa3; histone cluster 2, H2aa4	ENSEMBL:ENSP00000358155;ENSP00000358158
P84243	h3f3a	2736;8033;8668;10795	2662;7735;8347;10397	4;3;3;3;3;3;2;1	4;3;3;3;3;3;2;1	1;0;0;0;0;0;0;0	IPI00219038;IPI00171611;IPI00216402;IPI00465070;IPI00419884;IPI00909530;IPI00477080;IPI00784001	H3 histone, family 3B (H3.3B); H3 histone, family 3A pseudogene; H3 histone, family 3A; similar to H3 histone, family 3B; similar to histone H3.3B	ENSEMBL:ENSP00000254810;ENSP00000355778;ENSP00000355780;ENSP00000355781
[illegible]	[illegible]	[illegible]	[illegible]	[illegible]	[illegible]	[illegible]	[illegible]	[illegible]	[illegible]

Uniprot Acc	Proteins	Peptides	Razor + unique peptides	Unique peptides	Sequence coverage [%]	Unique + razor sequence coverage [%]	Unique sequence coverage [%]	Mol. weight [kDa]	Sequence length	Sequence lengths	PEP	Ratio H/L	Ratio H/L normalized	Ratio H/L normalized Significance A	Ratio H/L normalized Significance B	Ratio H/L variability [%]	Ratio H/L count	Gene Synonyms	Intensity	Intensity L	Intensity H
Q9H2U1-1	4	1	1	1	1,3	1,3	1,3	114,76	1008	1008;994;994;979	0,000868	0,39470	0,27485	6,35E-12	8,89E-12	113,29	3	DHX36	3433300	2043600	1389700
Q92542-2	7	2	2	2	4,5	4,5	4,5	76,743	689	689;709;571;451;320;305;219	2,90E-23	0,44611	0,27871	1,03E-11	1,42E-11	225,42	2	NCSTN	12718000	8173700	4544700
P05161	1	4	4	4	30,9	30,9	30,9	17,887	165	165	1,03E-57	0,43120	0,30060	1,34E-10	6,99E-14	12,585	8	ISG15	115970000	83075000	32896000
P09913	1	2	2	2	4,8	4,8	4,8	56,212	484	484	9,39E-10	0,46585	0,31566	6,52E-10	7,56E-10	9,7732	2	Ifit2	10724000	7294500	3429100
Q9ULE4	1	1	1	1	1,1	1,1	1,1	121,04	1060	1060	0,000394	0,49305	0,33996	6,43E-09	3,80E-08	9,8338	6	Fam184b	42914000	28929000	13984000
Q93096	1	1	1	1	8,7	8,7	8,7	19,815	173	173	0,000572	0,51387	0,35883	3,13E-08	3,14E-08	19,727	2	ptp4a1	2275400	1649200	626290
O75880	2	2	2	2	10	10	10	33,814	301	301;270	0,000732	0,58217	0,36827	6,52E-08	6,37E-08	25,459	2	sco1	3743700	1936300	1807400
P07203	1	2	2	2	19,7	19,7	19,7	22,041	203	203	9,76E-07	0,61702	0,40119	6,55E-07	5,92E-07	3,3184	2	GPX1	3966600	2757600	1209000
Q15392	2	1	1	1	3,7	3,7	3,7	60,101	516	516;475	2,25E-06	0,73681	0,46795	2,64E-05	2,14E-05	0,5743	2	DHCR24	4646100	2724100	1922000
Q99959-1	3	2	2	2	2,8	2,8	2,8	97,414	881	881;837;824	6,64E-16	0,83202	0,54360	0,000559603	0,000424217	4,8773	4	Pkp2	5384200	2978400	2405800
P07951-2	8	8	3	2	25	14,8	10,2	32,989	284	284;284;322;284;284;248;303;158	2,97E-107	0,77131	0,54591	0,000605455	0,00109041	12,915	8	tpm2	57581000	31508000	26073000
P17096-3 Tax_Id=9606 Gene_Symbol=HMGA1 Isoform HMG-R of High m	7	2	2	2	7,3	7,3	7,3	34,301	328	328;179;107;115;109;107;96	1,75E-10	0,84502	0,57045	0,00133392	0,000997273	14,242	2	HMGA1	13940000	7052100	6887900
O94888	2	3	3	3	7,8	7,8	7,8	54,862	489	489;75	4,54E-16	0,84338	0,57172	0,00138669	0,00230223	31,274	3	UBXN7	24396000	12503000	11892000
P21980-1	4	4	4	4	7,9	7,9	7,9	77,328	687	687;627;606;548	1,10E-33	0,86565	0,57753	0,00165139	0,00269546	7,5655	4	tgm2	32000000	18036000	13963000
O75044	5	2	2	2	2,1	2,1	2,1	120,88	1071	1071;1070;836;1085;1062	0,000142	0,91802	0,58092	0,00182499	0,00135861	49,727	2	srgap2	3551900	1928400	1623600
P11802	9	3	2	2	9,2	6,6	6,6	33,729	303	303;203;201;183;170;115;111;74;40	7,33E-05	0,84688	0,58903	0,00230539	0,00171127	253,25	2	Cdk4	11361000	9543600	1817800
P61254	2	1	1	1	5,9	5,9	5,9	18,254	153	153;145	6,02E-06	0,91735	0,59751	0,00291981	0,000898365	2,6749	2	RPL26	97264000	52289000	44975000
Q6FI13	2	6	1	1	60	22,3	22,3	14,095	130	130;129	1,60E-82	0,89000	0,59794	0,00295425	0,0045585	11,654	3	HIST2H2AA3	51117000	30846000	20271000
P84243	8	4	4	1	40,4	40,4	23,5	15,328	136	136;136;136;136;135;113;123;68	7,08E-46	0,96749	0,60901	0,00397131	0,00135095	11,594	5	h3f3a	283930000	152040000	131890000
[illegible]	[illegible]	[illegible]	[illegible]	[illegible]	[illegible]	[illegible]	[illegible]	[illegible]	[illegible]	[illegible]	[illegible]	[illegible]	[illegible]	[illegible]	[illegible]	[illegible]	[illegible]	[illegible]	[illegible]	[illegible]	[illegible]
